# Abdominal Visceral Adipose Tissue and All-Cause Mortality: A Systematic Review

**DOI:** 10.3389/fendo.2022.922931

**Published:** 2022-08-22

**Authors:** Randa K. Saad, Malak Ghezzawi, Renee Horanieh, Assem M. Khamis, Katherine H. Saunders, John A. Batsis, Marlene Chakhtoura

**Affiliations:** ^1^ Calcium Metabolism and Osteoporosis Program, World Health Organization (WHO) Collaborating Center for Metabolic Bone Disorders, Division of Endocrinology and Metabolism, Department of Internal Medicine - American University of Beirut Medical Center, Beirut, Lebanon; ^2^ Departement of Internal Medicine, American University of Beirut Medical Center, Beirut, Lebanon; ^3^ Wolfson Palliative Care Research Centre, Hull York Medical School, University of Hull, Hull, United Kingdom; ^4^ Division of Endocrinology, Diabetes & Metabolism, Weill Cornell Medicine, New York, NY, United States; ^5^ Division of Geriatric Medicine and Department of Nutrition, University of North Carolina at Chapel Hill, Chapel Hill, NC, United States

**Keywords:** systematic review, visceral adipose tissue, abdominal visceral fat, fatal outcome, all-cause mortality

## Abstract

**Introduction:**

Increased abdominal visceral adipose tissue (VAT) implies an adverse cardio-metabolic profile. We examined the association of abdominal VAT parameters and all-cause mortality risk.

**Methods:**

We systematically searched four databases. We performed citations/articles screening, data abstraction, and quality assessment in duplicate and independently (CRD42020205021).

**Results:**

We included 12 cohorts, the majority used computed tomography to assess abdominal VAT area. Six cohorts with a mean age ≤ 65 years, examining all-cause mortality risk per increment in VAT area (cm^2^) or volume (cm^3^), showed a 11-98% relative risk increase with higher VAT parameters. However, the association lost significance after adjusting for glycemic indices, body mass index, or other fat parameters. In 4 cohorts with a mean age >65 years, the findings on mortality were inconsistent. Conversely, in two cohorts (mean age 73-77 years), a higher VAT density, was inversely proportional to VAT area, and implied a higher mortality risk.

**Conclusion:**

A high abdominal VAT area seems to be associated with increased all-cause mortality in individuals ≤ 65 years, possibly mediated by metabolic complications, and not through an independent effect. This relationship is weaker and may reverse in older individuals, most likely secondary to confounding bias and reverse causality. An individual participant data meta-analysis is needed to confirm our findings, and to define an abdominal VAT area cutoff implying increased mortality risk.

**Systematic Review Registration:**

https://www.crd.york.ac.uk/prospero/display_record.php?RecordID=205021, identifier CRD42020205021.

## Introduction

Obesity is currently defined as a body mass index (BMI) ≥ 30 kg/m^2^ (1); a cutoff that has been linked to increased mortality in Caucasians (2). However, BMI does not take into consideration body fat and lean mass distribution, that might differentially impact the health risks associated with excess weight (3, 4). While both abdominal subcutaneous adipose tissue (SAT) and visceral adipose tissue (VAT) imply an increased morbidity, the association remains stronger with VAT (5, 6). VAT represents increased ectopic fat deposits in metabolically important organs, including liver and pancreas (7). It plays a role in the pathophysiology of cardio-metabolic diseases, by releasing fatty acids into the circulation, secreting adipokines in a poorly regulated manner (5), and therefore resulting in a subclinical inflammatory state (4). Abdominal VAT constitutes a risk factor for cardiovascular (CV) diseases (8), cancer and stroke (9). However, the association with all-cause mortality has been inconsistent (10–12).

Waist circumference and waist-to-hip ratio measurements reflect abdominal obesity and VAT (13–16). However, imaging modalities, such as computed tomography (CT), magnetic resonance imaging (MRI) and dual-energy x-ray absorptiometry (DXA), allow a more accurate assessment of ectopic fat at specific sites (4, 17–20). CT and MRI directly measure VAT parameters, whereas DXA VAT measurement is derived from the difference between total fat and SAT measurements (4). DXA has been validated against other modalities (20, 21), and showed a high accuracy. Given its lower cost and lower radiation compared to CT and MRI, DXA can be used for serial follow up of VAT (19).

The aim of this systematic review (SR) is to examine the association between abdominal VAT parameters, evaluated by imaging, and the risk of all-cause mortality.

## Methods

We followed the Preferred Reporting Items for Systematic Reviews and Meta-Analyses (PRISMA) Guidelines (**Appendix 1**). The protocol for this SR is published on PROSPERO (CRD 42020205021) (22).

### Literature Search

With the help of a medical librarian (LH), we performed a systematic search in Medline, Embase, CINAHL, and the Cochrane library, from inception until May 2022. We used MeSH terms and keywords related to abdominal or intra-abdominal obesity or fat, adipose tissue, body composition, MRI, CT, densitometry, myocardial infarction, stroke or brain infarction, unstable angina, cerebrovascular disorders, and mortality (see **Appendix 2A** for search details). We also screened the reference lists of included studies and reviews on the topic. We did not limit to specific languages but excluded studies published in Japanese or Mandarin.

### Selection of Studies

Pairs of two reviewers (RS, MG, RH) screened citations and abstracts, and full texts, in duplicate and independently, using an *a priori* prepared screening guide. Reviewers conducted a calibration exercise, by piloting a sample of 20 abstracts and full text articles, and by comparing their results. If discrepancy rate was > 5%, the reviewers examined the discordant documents, and with the advice of a content expert (MC), modified the screening guide to ensure standardization of the screening process. Reviewers repeated the calibration exercise until achieving >95% concordance rate.

#### Included Studies

We planned to include observational (cohorts and nested case control studies) and interventional studies, when available, extending over at least one year, in order to have a complete summary of the evidence on the topic. We excluded case reports, case series, cross sectional studies, and narrative/systematic reviews.

The population of interest consisted of adult participants (≥ 18 years old). We excluded studies where > 25% of participants have diseases known to affect body composition, such as endocrinopathies, malnutrition, parenteral nutrition, cachexia, trauma or stress associated conditions, drug-injection associated conditions, individuals positive for HIV, previous abdominal laparotomy or bariatric surgery, autoimmune diseases, partial or generalized lipodystrophy, and chromosomal abnormalities or single gene defects. We also excluded studies where > 25% of the participants were pregnant women, or patients with baseline CV, cerebrovascular, advanced kidney or liver disease, or who have a solid or a hematological malignancy or have undergone a solid organ transplantation (23).

We included studies that measured any abdominal VAT parameter [area (cm^2^), volume (cm^3^), mass (g) or density (g/cm^3^)], using MRI, CT scan or any of the United States (US) Food and Drug Administration approved DXA machines (Lunar, Hologic, Norland), and excluded those using bioelectric impedance.

The outcome of interest was all-cause mortality. We included any study reporting on all-cause mortality as a main study outcome or as an adverse event. Studies on CV outcomes are beyond the scope of this paper (Refer to **Appendix 2B** for a full details of the excluded studies).

### Data Extraction

Pairs of two reviewers (RS, MG, RH) performed data abstraction in duplicate and independently, on population baseline characteristics, details of abdominal VAT measurements, and outcome of interest. We contacted corresponding authors *via* email in case of missing data. We assessed the risk of bias (ROB) in duplicate and independently, using the Newcastle-Ottawa Quality Assessment Scale (NOS) (24) for observational studies, and planned to use the Cochrane Collaboration tool for trials, but did not identify any (25). For ROB assessment, we also performed a calibration exercise among reviewers until reaching a concordance rate of > 95%.

We used SPSS version 23 (IBM, Chicago, USA) for graphical representation of hazard ratios (HR) or odds rations (OR) of various studies. We rounded numbers to the nearest decimal, unless otherwise stated.

## Results

### Search Results

**Figure 1:**
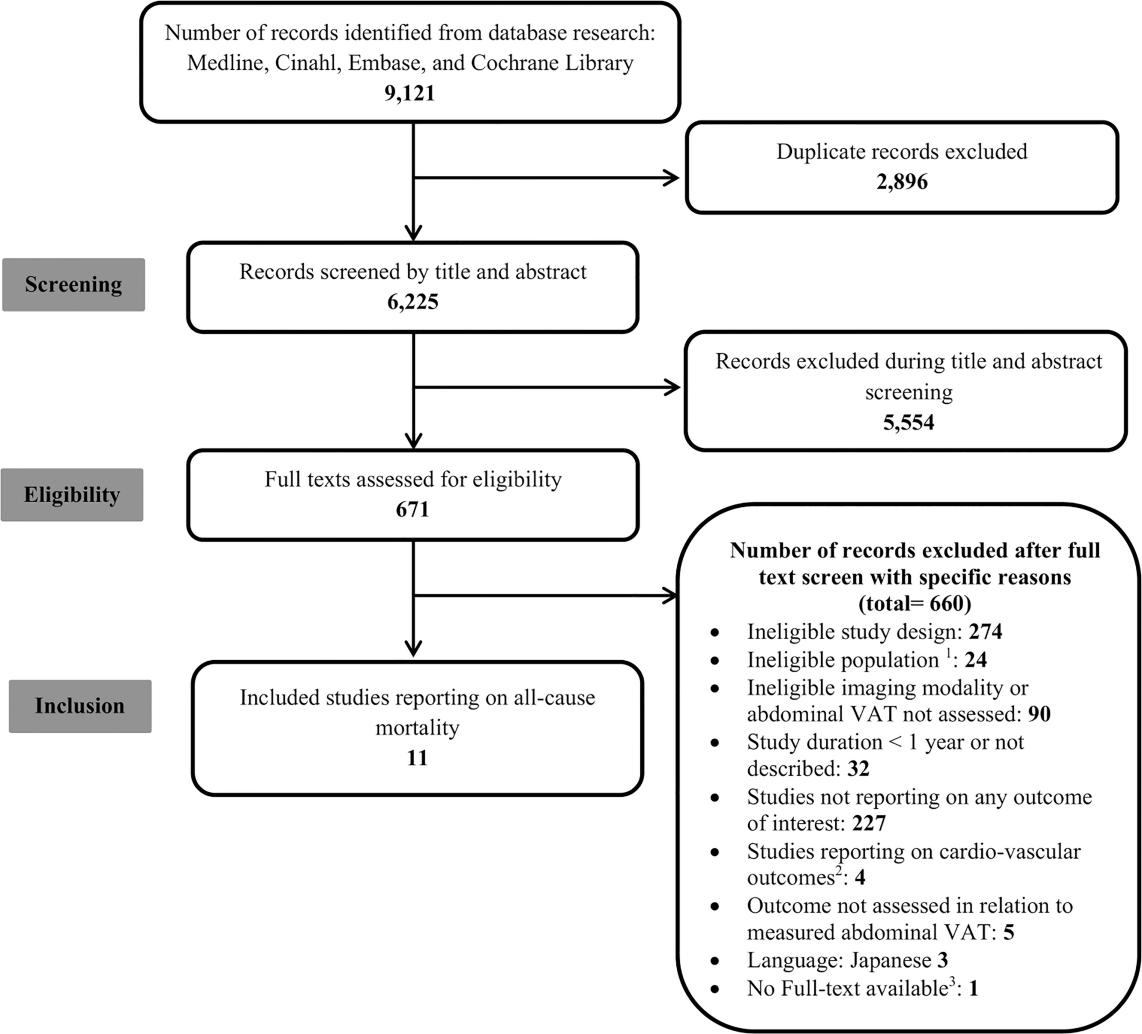
Flow Diagram. ^1^Age < 18; pregnant women; baseline cardiovascular, cerebrovascular, chronic kidney or cirrhotic liver disease; malignancy; transplant; Human Immunodeficiency Virus infection; abdominal surgery; conditions associated with abnormal fat distribution (lipodystrophy, malnutrition, parental nutrition, endocrinopathies, autoimmune conditions, drug-injection, trauma or stress, and chromosomal abnormalities); ^2^ Cardio-vascular outcomes are eligibile as per our registered protocol but are beyond the scope of the current manuscript; ^3^ We were not able to find the full text even after seeking help from a medical librarian.

We identified 6225 citations after duplicate removal, of which we screened 671 full texts. We included 11 articles, with data from 12 cohorts, evaluating the association between VAT and all-cause mortality (10, 11, 26–34) (**Figure 1**). We did not identify any interventional study.

### Characteristics of Included Studies

The majority of studies (6/11) were cohorts based in the US (10, 11, 26, 27, 31, 32), including the Multi-Ethnic Study of Atherosclerosis (MESA) cohort (26), the Health, Aging, and Body Composition (Health ABC) cohort (27), the Pennington Center Longitudinal Study (31), and a sub-population of the Framingham Heart Study Offspring and Third Generation cohorts (11). Two cohorts were derived from the Age, Gene and/or Environment Susceptibility-Reykjavik (AGES-Reykjavik) study from Iceland (27, 30), two from South Korea (28, 29), and one cohort each from Brazil (33) and Sweden (34). The sample size varied between 291 and 34,080 participants (**Table 1**).

**Table 1:** Summary of the characteristics of the included studies reporting on the association of visceral adipose tissue and all-cause mortality^1^.

Author, Country, Study Period	PopulationSampling Method	Sample Size	Age (years) Mean (SD)	Women (%)	Ethnicity (%)	BMI (Kg/m^2^)Mean (SD)	Smoking (%)	Imaging Modality & Anatomical Landmark	Physical Activity Assessment	Mortality Data Source	Follow-up (years)Mean (SD)	Study Quality
Britton ^(11)^ US 2002-2005	Framingham Heart Study Offspring and Third Generation cohorts, free of CVD and cancer, and with complete covariate data who underwent MDCT	3,086	50.2 (10.0)	49.0	White**^2^** 98.4	27.7 (5.2)	Current: 12.5 Former: 38.2	CT 125 mm above the level of S1	No	NA	Median 5.0 (IQR 3.9-6.0)	Good 4-2-2
Chung ^(29)^ South Korea 2007-2015	Patients enrolled for a comprehensive health checkup at Seoul National University Hospital Healthcare System Gangnam Center	34,080	51.4 (9.8)	41.4	NA	23.6 (3.1)	NA	CT Umbilical level	No	Korea National Statistical Office	6.9 (2.7)	Good 4-2-2
De Santana ^(33)^ Brazil 2005-2012	Well-functioning older Butantã district residents from 66 randomly selected census sectors	839	73.2 (5.3)	61.5	Caucasian 64.7	27.9 (5.0)	Current: 11.6	DXA VAT estimated from the android region**^3^**	Y es**^4^**	PRO‐AIM, organ responsible for vital statistics, operating under the auspices of the São Paulo State Secretary of Economics and Planning	4.1 (1.1)	Good 4-2-3
Shil Hong ^(28)^ South Korea 2005-2011	Ages ≥ 65 from stratified random sample in the Korean Longitudinal Study on Health and Aging (KLoSHA)	1,000	76.0 (8.7)	56.1	NA	23.9 (3.4)	Current: 11.7 Former: 26.9	CT Umbilical level	Yes**^5^**	Korean National Statistical Office	Median 5.2 (IQR 0.1-6.3)	Good 4-2-3
Katzmarzyk ^(31)^ US 1995-2009	The Pennington Center Longitudinal Study (PCLS); Volunteers participating in clinical studies at the PBRC in Baton Rouge, Louisiana and had undergone a CT scan of the abdomen	1,089	46.0 (12.5)	55.0	White 100	29.7 (5.2)	NA	CT L4/L5	No	National Death Index	9.1 (3.3)	Good 4-2-2
Koster ^(30)^ Iceland 2002-2013	AGES-Reykjavik study; Random sample of a cardiovascular cohort that begun in 1967 to study heart disease	5,087	76.4 (5.5)	57.0	NA	27.1 (4.2)	Current: 9.2 Former: 43.4	CT L4/L5	Yes^6^	Icelandic National Roster	8.0 (NA)	Good 4-2-2
Kuk ^(10)^ US 1995-1999	Cohort who received a preventive medicine diagnostic exam at the Cooper Clinic in Dallas, Texas; Cases: selected from cohort receiving CT examination of the abdomen as part of a preventive medicine diagnostic exam; Controls: randomly selected from survivors, alive at the time of death of the decedent	291	56.4 (12.0)	0	White 98.0	26.7 (3.8)	Current: 12.4 Former: 29.9	CT L4-L5 and L3-L4	No	Official death certificates	2.2 (1.3)	Fair 2-2-1
McNeely ^(32)^ US 1983-2007	Staggered enrollment of 2^nd^ generation (Nisei) and 3^rd^ generation (Sansei) men and women of 100% Japanese ancestry	733	Range**^7^**34.0-74.0 (NA)	NA	Japanese 100	NA	NA	CT Umbilical level	No	National Death Index	16.9 (NA)	Good 4-2-3
Mongraw-Chaffin ^(26)^ US 2002-2013	30% random subsample without previous CVD from all 6 US areas of Multi-Ethnic Study of Atherosclerosis (MESA) sites	1,886	Range 63.6-66.0 (0.37-0.39)	50.0	White 40.0 Asian 13.0 African American 21.0 Hispanic 26.0	24.6-31.4 (0.16-0.19)	Current: 8.3-14	CT L4/L5	Yes	Death certificates, medical records, autopsy reports, interviews with participants, and, in the case of out-of-hospital deaths, interviews with or questionnaires to physicians, relatives, or friends	9.3 (NA)	Good 4-2-2
Murphy ^(27)^ US & Iceland 1997-2011 ^(13,30)^ **^8^**	Community dwelling well-functioning participants from the Health ABC study ^(13)^; Random sample of white Medicare beneficiaries and all black Medicare eligible residents in Memphis, Tennessee, and Pittsburgh, Pennsylvania	2,735	Range Women: 73.2-74.0 (2.7-3.1) Men: 73.4-74.0 (2.8-2.9)	50.8	Black Women: 43.6-44.0 Men: 36.1-36.2	Women: 23.2-30.1 (4.1-5.1) Men: 23.9-29.3 (3.0-3.7)	Current: Women 5.7-14.2 Men 7.8-19 Former: Women 23.6-40.1 Men^9^ 50-69	CT L4/L5	Yes**^10^**	Death certificates, hospital records, and interview with next of kin	Total 14	Good 4-2-2
	Single center population-based participants from the AGES-Reykjavik study ^(31)^; Random sample of a cardiovascular cohort that begun in 1967 to study heart disease	5,131	Range Women: 75.5-77.1 (5.0-6.2) Men: 76.0-77.5 (5.0-5.6)	57	NA	Women: 23.1-30.5 (3.4-4.7) Men: 23.6-29.5 (3.0-3.6)	Current: Women 12-15.4 Men 7.7-19.3 Former: Women 29.8-36.9 Men 49.7-67.6	CT L4/L5	Yes**^11^**	Icelandic National Roster	Range 4-9	
Ballin ^(34)^ Sweden 2012-2018	This study was based on the HAI, which is a population- based prevention study conducted at a single research clinic in Umeå, Sweden. Population composed of community-dwelling residents of Umeå and exact age of 70 years, with no exclusion criteria. Eligible individuals were identified and invited using population registers.	3,294	70.4 (0.1)	49.6	NA	26.3 (4.0)	5.7	DXA android region	Yes^12^	Swedish Cause of Death Register, which (maintained by the Swedish National Board of Health and Welfare)	3.6 (range, 0.1– 6.6)	Good 4-2-3

AGES-Reykjavik, The Age, Gene and/or Environment Susceptibility-Reykjavik; BMI, Body mass index; CT, Computed tomography scan; CVD, Cardiovascular disease; DXA, Dual-energy x-ray absorptiometry; Health ABC, The Health, Aging, and Body Composition; HAI, Healthy Ageing Initiative; IQR, Interquartile range; MDCT, Multidetector computed tomography; NA, Not available; PBRC, Pennington Biomedical Research Center; PRO‐AIM, Programa de Aprimoramento das Informações de Mortalidade no Município de São Paulo.

^1^Continuous variables are expressed as means (SD) unless otherwise specified. ^2^Splansky et al. The Third Generation Cohort of the National Heart, Lung, and Blood Institute's Framingham Heart Study: Design, Recruitment, and Initial Examination. July 2007. American Journal of Epidemiology 165(11):1328-35. ^3^The inferior line of this region is drawn just at the superior edge of the iliac crest, whereas the superior line is at 20% of the distance between the iliac crest and the inferior edge of the chin. VAT results from the subtraction of the subcutaneous android fat from the total android fat. ^4^Low physical activity score. ^5^Regular exercise. ^6^Hours per week of moderate to vigorous physical activity. ^7^Study excluded death after the age of 82 years from the analysis. ^8^Data derived from Murphy population (Health ABC and AGES-Reykjavik population). ^9^ >100 cigarettes in a lifetime. ^10^Activity 7 days prior to baseline. ^11^Frequency of moderate to vigorous activity 1 year prior to baseline. ^12^Moderate- to- vigorous physical activity was measured during 1 week using hip- mounted Actigraph GT3X+ accelerometers, and was 33.1±25.7 minutes per day.

One study exclusively included men (10), while women constituted 41-62% of the rest of the cohorts (11, 26–31, 33, 34). The major ethnicity was white for five of the cohorts from the US (10, 11, 26, 27, 31), and Caucasian, Black and Japanese ethnicities were each represented in one study (**Table 1**). The mean population age in half of the studies ranged between 46.0 and 56.4 years (10, 11, 29, 31), while the other half constituted an older population with a mean age range of 63.6-66.0 years in one study (26) and 70.3 to 77.5 years in four other cohorts (27, 28, 30, 33, 34). Participants were classified as having overweight or obesity, with a mean BMI ≥ 25.0 kg/m^2^ for studies from the US, Europe or Brazil (10, 11, 30, 31, 33), and ≥23 kg/m^2^ for South Korean studies (28, 29), or mean BMI 23-31 kg/m^2^ (**Table 1**). As for co-morbid conditions (**Appendix 3**), one study included 38% of patients with non-alcoholic fatty liver disease (29), and all cohorts included participants with diabetes mellitus (DM), hypertension and dyslipidemia, with various proportions. Two cohorts included a small proportion (< 25%) of participants with CV events at baseline (28, 34). Six studies reported on the percent of individuals who consume alcohol, ranging from 15 to 50% (27–31, 33, 34).

The imaging modality used was CT scan for 9/11 studies, performed at L1, L4-L5, or at the umbilical level (10, 11, 26–32). Two studies used DXA, and the VAT area was estimated from the android region (33, 34). None of the studies used MRI (**Table 1**). Mortality was ascertained either through a national database or official death certificates and/or hospital records (**Table 1**).

The funding was described in all studies, and the conflict of interest was reported in 9/11 (**Appendix 4A**). Our ROB assessment showed that all studies were of good quality, with the exception of one fair quality study (**Table 1**, detailed ROB assessment in **Appendix 4B**).

### Abdominal VAT Area (cm^2^) and All-Cause Mortality

Two studies (women 50-56%) reported on the association of VAT area categories and all-cause mortality (26, 28). The population-based Korean Longitudinal study on Health and Aging (KLoSHA) cohort (N=1,000, mean BMI 23.9 ± 3.4 kg/m^2^), included an older population (mean age 76.0 ± 8.7 years) and followed patients for a median of 5.2 (range 0.1-6.3) years (28). It reported that the HR of all-cause mortality significantly decreases by 44-68% with increasing abdominal VAT area quartiles, compared to the smallest VAT area quartile (VAT area= 7-74cm^2^) as a reference, and after adjusting for age, sex, alcohol consumption, smoking status, exercise habit, and total fat mass, p for trend 0.001 (28). Conversely, the MESA cohort (N=1,910, mean BMI 28.1 ± 2.9 kg/m^2^), enrolling a younger population (mean age 64.6 ± 1.2 years) reported an increased risk of all-cause mortality with a VAT area ≥ 171.9 cm^2^, compared to < 110.9 cm^2^, at a mean follow up of 9.3 years (26). However, the association lost statistical significance after adjusting for age, sex, ethnicity, education, income, smoking and subcutaneous fat (26).

Eight studies reported on the risk (HR or OR) of all-cause mortality, per increase in VAT area (cm^2^) (10, 26, 28–33), defined as the population specific VAT area standard deviation (SD), interquartile range (IQR), or a pre-specified VAT value, depending on the study. Therefore, given the heterogeneity in the reporting of results in individual studies, we were unable to pool them in a meta-analysis.

**Figure 2:**
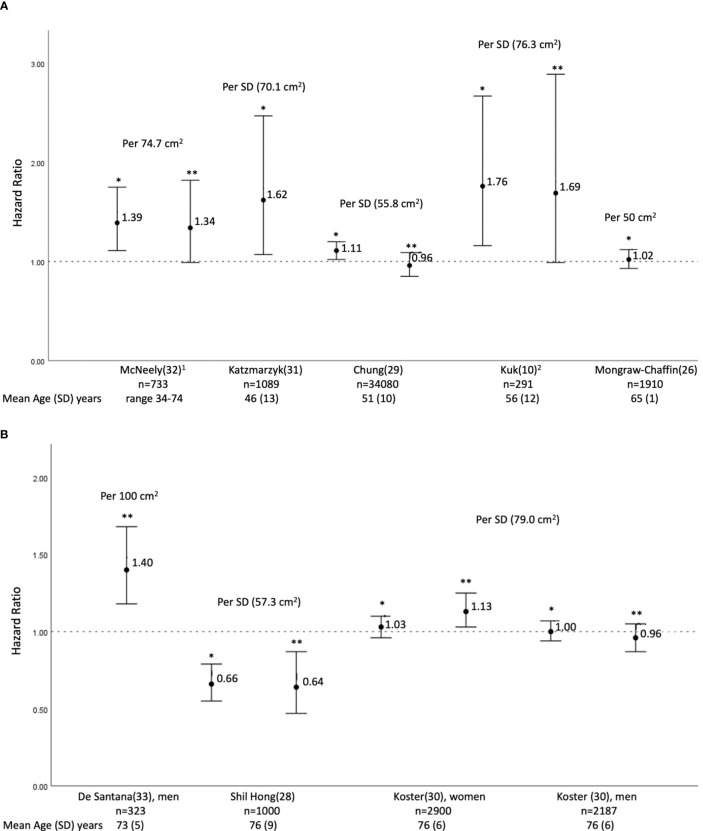
Hazard Ratios (HRs) and 95% confidence interval of all-cause mortality per increase in abdominal visceral adipose area (cm^2^); the increase being defined individual studies as the standard deviation or as a fixed number. **(A)** studies with participants’ mean age of 65 years or less; **(B)** studies with participants’ mean age above 65 years. ^1^ Result reported as hazard ration (HR) per incremental increase in abdominal visceral adipose tissue equivalent to the magnitude of the interquartile range (IQR), 25^th^ percentile 48.2 cm^2^ and the 75^th^ percentile 122.9 cm^2^ (personal communication with author). ^2^ Mortality risk expressed as Odds ration (OR). *Unadjusted or adjustment does not include BMI and glycemic parameters; McNeely et al (32) HR adjusted for sex and smoking. Katzmarzyk et al (31) ) HR adjusted for age, sex, smoking, alcohol consumption, exam year, subcutaneous tissue area, physical activity and excluding patients with a history of stroke, heart disease and cancer at baseline. Chung et al. (29) unadjusted HR. Koster et al (30) HR adjusted for age, education, smoking, physical activity , and alcohol. ** Adjusted includes BMI and/or glycemic parameters; McNeely et al. (32) HR adjusted for sex, smoking and BMI. Chung et al. (29) HR adjusted for age, sex, diabetes, hypertension, fatty liver. Subcutaneous tissue area, and significant alcohol consumption. Kuk et al. (10). OR adjusted for age, follow-up time, abdominal subcutaneous fat and liver fat. De Santana et al. (33) OR adjusted for age, low level of physical activity, recurrent falls, diabetes mellitus, hypertension, previous cardiovascular event, serum phosphorus, serum calcium, albumin and total hip bone mineral density T-score. Shil Hong et al (28) HR adjusted for age, sex, smoking, alcohol consumption, physical activity, and total fat mass. Koster et al. (30) HR adjusted for age, education, smoking, physical activity, alcohol, body mass index, type II diabetes, and coronary heart disease.

Five of the eight cohorts enrolled younger participants, with a mean age ≤ 65 years (10, 26, 29, 31, 32) (**Figure 2A**). There was an increase in the risk of all-cause mortality with increased VAT parameters in four of the studies (10, 29, 31, 32), but the association lost statistical significance in three of them after adjusting for BMI, glycemic parameters, or other fat parameters (10, 29, 32). A cohort of participants of Japanese ancestry from the US (N=733, age range 34-74 years) reported a 39% greater risk of all-cause mortality per IQR (74.7 cm^2^) increase in abdominal VAT area, aHR 1.39 (95% CI 1.11–1.75) (32). This, however, became non-significant after further adjustment for BMI or glycemic indices (32). A large cohort from South Korea (N=34,080, mean age of 51.4 ± 9.8 years, 41% women), showed a higher mean VAT area in subjects who were deceased, compared to those who survived, at a mean follow-up of 6.9 ± 2.7 years (29). In the univariate model, an increase in VAT area was significantly associated with a higher risk of overall mortality (HR 1.11, 95% CI 1.02–1.20, per SD (55.8 cm^2^) of VAT area). However, the analysis adjusting for DM, hypertension, fatty liver and other predictors showed no association (29). This was not different when individuals with overweight/obesity (BMI ≥ 25 kg/m^2^) and those without (BMI < 25 kg/m^2^) were analyzed separately, nor when patients with significant alcohol consumption were excluded (29). A cohort of volunteers presenting for clinical studies at the Pennington Biomedical Center (N=1,089, youngest population, mean age 46.0 ± 12.5 years, 54% women) showed a 62% increase in all-cause mortality per SD (70.1 cm^2^) increase in VAT area at an average of 9.1 years of follow up (aHR 1.62, 95% CI 1.07–2.47), adjusting only for demographics and social history (31). The latter study did not investigate the confounding effect of glycemic or metabolic parameters (31). The cohort with the shortest follow up (mean 2.2 ± 1.3 years) included men from a single center in Texas (N=291, mean age 56.4 ± 12.0 years), and showed a significantly higher VAT area in decedents, compared to participants who were alive, after adjusting for age and follow up length (10). When several fat parameters were included in the same model, there was no association of visceral fat area, subcutaneous fat area and liver fat, with all-cause mortality (10). The MESA cohort (N=1,910, mean age 64.6 ± 1.2 years) reported no significant risk per 50 cm^2^ increase in abdominal VAT area, aHR 1.02 (95% CI 0.93–1.12) (26). Noteworthy, a major limitation of the MESA cohort, as recognized by the investigators, was the high proportion of missing data from participants at the upper end of visceral fat measurement.

Results of the three studies in older individuals (mean age > 65 years) were inconsistent (28, 30, 33) (**Figure 2B**). The AGES-Reykjavik cohort (N=5,087; mean age 76 ± 5.5 years) showed no increase in all-cause mortality, per SD (79.0 cm^2^) increase of VAT area, at an average follow up of eight years, aHR 1.04 (95% CI 0.89, 1.22) (30). Sex-specific analysis showed no association in men (aHR 0.96, 95% CI 0.87–1.05), but a 13% increased risk in women with higher VAT (aHR 1.13, 95% CI 1.03–1.25), even after adjustment for BMI, DM and heart disease (30). The Brazilian cohort (N= 839; mean age 73.2 ± 5.2 years, 62% women, 63% with hypertension at baseline) reported a 40% increase in all-cause mortality (aOR 1.40, 95% CI 1.18-1.68) per 100 cm^2^ increase in VAT area in men but not in women, adjusting for DM and other cardio-metabolic parameters (33). Conversely, the population-based KLoSHA cohort (N= 1,000; mean age 76 ± 8.7 years, 56% women) reported a 36% lower risk of all-cause mortality per SD of VAT area (57.3 cm^2^), over a follow-up period of up to six years, aHR 0.64 (95% CI 0.47-0.87). The KLoSHA cohort did not investigate the confounding effect of glycemic parameters (28).

### Abdominal VAT Density (g/cm^2^) and All-Cause Mortality

Two cohorts (women 51-57%, mean age 73-77 years) reported on the association of VAT density quintiles with all-cause mortality, the Health ABC cohort (N= 2,735, mean BMI 27.2 ± 4.6 kg/m^2^) and the AGES-Reykjavik cohort (N= 5,131, mean BMI 27.0 ± 4.4 kg/m^2^) (27). These studies followed up participants for a total of 14 years, and a range of 4-9 years, respectively (27). Both cohorts reported an increased risk of mortality of women in the VAT quintile with the highest density (higher fatty infiltration), compared to the least dense VAT quintile, HR 1.72 (1.19–2.48) and 1.65 (1.15–2.36), respectively for Health ABC and AGES-Reykjavik, after adjustment for age, education, BMI, fat area of respective fat depot, sagittal diameter, smoking status, drinking status, physical activity, comorbid conditions, weight history (% change from midlife) and prior hospitalization (27). The AGES-Reykjavik additionally adjusted for time of computed tomography scan, while the Health ABC additionally adjusted for race and study site (27).

**Figure 3:**
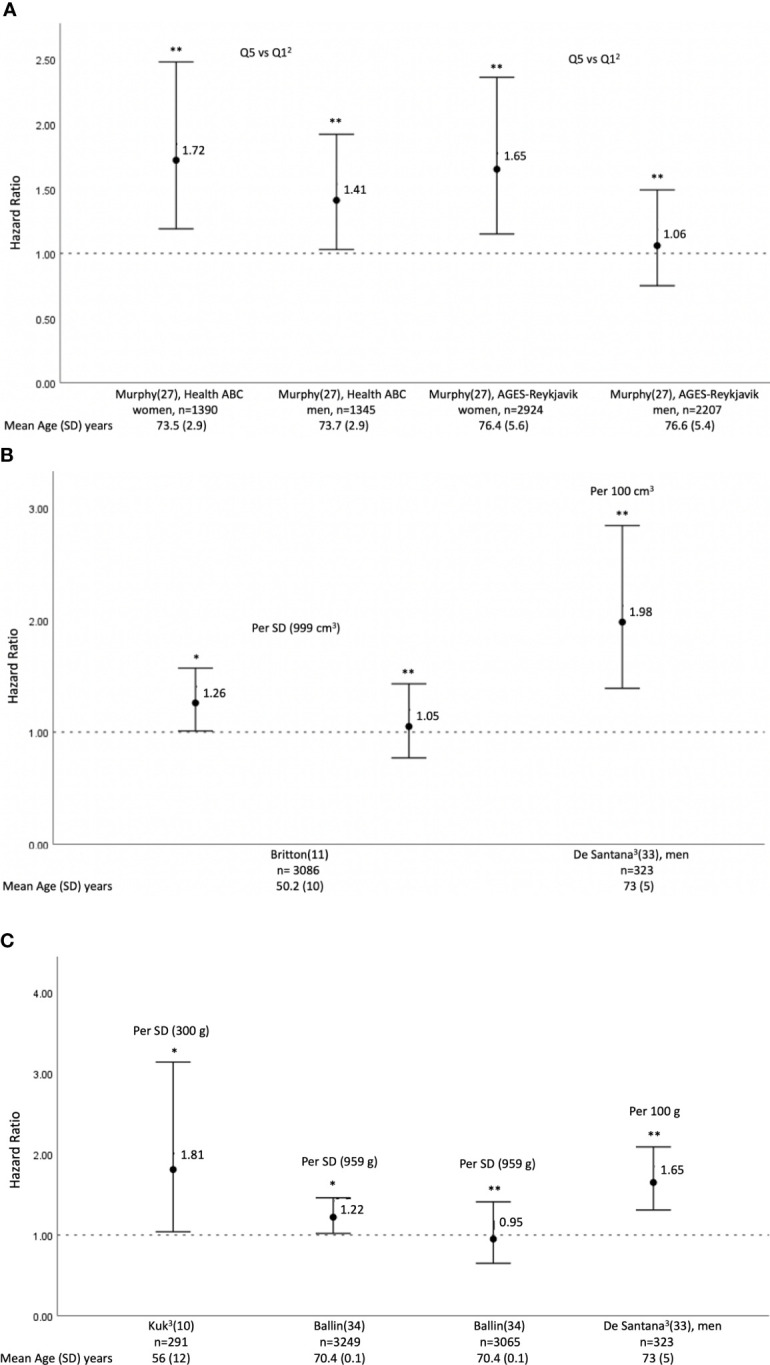
Hazard Ratios^1^ (HRs) an 95% confidence interval of all-cause mortality per increase in abdominal visceral adipose **(A)** density^2^(g/cm^2^), **(B)** volume (cm^3^), **(C)** mass (g). The increase in each parameter being defined in individual studies as the standard deviation of the parameter considered or as a fixed number. ^1^Result reported as hazard ratio (HR), unless indicated otherwise. ^2^Result reported as the most dense abdominal visceral adipose tissue quintile Q5, compared to the least dense visceral adipose tissue quintile Q1. 3Odds ratio (OR). * Unadjusted or adjustment does not include BMI and glycemic parameters; Britton et al. (11) HR adjusted for age and sex. Kuk et al. (10) OR adjusted for age, follow-up time, abdominal subcutaneous fat, and liver fat. Ballinet al. (34) unadjusted HR. ** Adjustment includes BMI and/or glycemic parameters; Murphy et al. (27), AGES-Reykjavik: HR adjusted for age, education, body mass index, area of respective fat depot, sagittal diameter, smoking status, drinking status, physical activity, comorbid conditions, time of computed tomography scan, weight history (% change from midlife) and prior hospitalization. Murphy et al. (27), Health ABC: HR adjusted for age, race, study site, education. body mass index, fat area of respective fat depot, sagittal diameter, smoking status, drinking status, physical activity, comorbid conditions. weight history (% change from midlife) and prior hospitalization. Britton et al. (11) HR adjusted for age, sex, systolic blood pressure, diabetes, total cholesterol, high-density lipoprotein cholesterol, current smoking, hypertension treatment, and body mass index. De Santana et al. (33) OR adjusted for age, low level of physical activity, recurrent falls, high alcohol intake, diabetes mellitus, previous cardiovascular event, serum phosphorus, calcium, albumin, 25‐OH Vitamin D and total hip BMD T‐score. OR for women not provided in paper. Ballinet al. (34) HR adjusted for sex, smoking, alcohol consumption, education, income, marital status, total fat mass, low-density lipoprotein cholesterol, fasting blood glucose, systolic blood pressure, previous stroke/MI/angina pectoris, prescribed antihypertensives/anticoagulants/lipid-lowering agents, and moderate-to-vigorous physical activity and muscle density.

Conversely, in men, only the Health ABC cohort reported an increased mortality associated with the VAT quintile with the highest density, compared to the least dense quintile, HR 1.41 (1.03–1.92), after adjusting for age, race, study site, education, BMI, fat area of respective fat depot, sagittal diameter, smoking status, drinking status, physical activity, comorbid conditions, weight history (% change from midlife) and prior hospitalization (27) (**Figure 3A**).

### Abdominal VAT Volume (cm^3^) and All-Cause Mortality

The Framingham Heart Study Offspring and Third Generation cohort (N= 3,086, mean age 50 ± 10 years, 49% women) assessed the correlation of VAT volume (cm^3^) with all-cause mortality (11). The results showed a 26% increased risk of all cause-mortality per SD increment in abdominal VAT volume (cm^3^), aHR 1.26 (95% CI 1.01-1.57), at a median follow-up of 5.0 years (IQR 3.9-6.0), adjusted for age and sex (11). The association lost significance when further adjustment for DM and hypertension was considered (11).

The Brazilian cohort reported a two-fold increase in all-cause mortality (aOR 1.98, 95% CI 1.38-2.84) per 100 cm^3^ increase in VAT mass in men but not in women, after adjusting for DM and other cardio-metabolic parameters (33) (**Figure 3B**).

### Abdominal VAT Mass (g) and All-Cause Mortality

The Brazilian cohort reported a 65% increase in all-cause mortality (aOR 1.65, 95% CI 1.31-2.09) per 100 g increase in VAT mass in men but not in women, after adjusting for DM and other cardio-metabolic parameters (33).

The cohort with the shortest follow up (mean 2.2 ± 1.3 years) only including men from a single center in Texas (N=291, mean age 56.4 ± 12.0 years) showed that, when several fat parameters were included in the same model, there was an 81% increase in all-cause mortality per SD increment in abdominal VAT mass (g), aOR 1.81 (95% 1.04-3.14) the association remained significant, even after adjusting for cardio-metabolic parameters (exact OR is not provided in the paper) (10). However, even though the Swedish study (N= 3,294) in an older population (mean age 70.4 ± 0.1 years) also reported a 22% increased risk of mortality per SD (959g) increase in VAT mass (g) (HR 1.22, 95% CI, 1.02– 1.46), this association lost statistical significance (aHR, 0.95, 95% CI, 0.65– 1.41) after adjusting for sex, smoking, alcohol consumption, education, income, marital status, total fat mass, low- density lipoprotein cholesterol, fasting blood glucose, systolic blood pressure, previous stroke/MI/angina pectoris, medications including antihypertensive, anticoagulants, and lipid- lowering agents, and moderate- to- vigorous physical activity and muscle density (34) (**Figure 3C**). This remained true in a sensitivity analysis in which early incident cases and participants with a short follow- up time and previous CVD were excluded.

## Discussion

Our SR of cohort studies showed that the VAT area was the most common parameter investigated in association with all-cause mortality and a higher abdominal VAT area was associated with an increased risk of all-cause mortality, with possible effect modification by age. Findings were consistent in studies enrolling individuals with a mean baseline age ≤ 65 years, and the risk may have been mediated by cardio-metabolic risk factors, specifically BMI, glycemic indices and fatty liver (10, 11, 29, 31, 32). Findings derived from studies enrolling older participants were inconsistent (27, 28, 30, 33) with some data suggesting a reversed relationship (27, 28). The effect modification by sex was variable across studies. Findings on VAT area volume and density were very heterogeneous.

Observational studies, mostly cross-sectional, showed that higher VAT parameters are associated with an increased risk of various cardio-metabolic risk factors, including DM (35, 36), dyslipidemia (37, 38), and hypertension (39–41), in both younger and older individuals; risk that seemed independent of insulin resistance, BMI, and waist circumference (35, 39, 41). Furthermore, recent data showed that mental illnesses, such as depression and schizophrenia, are associated with increased VAT, independent of weight, and this significantly impacts the risk of metabolic diseases in this population (42–44). Several pathways illustrate the mechanisms through which abdominal VAT implies a negative effect on the metabolic profile. VAT releases inflammatory proteins such as resistin (45), tumor necrosis factor-α (TNF-α), Interleukin-6 and C-reactive protein, and is associated with a decrease in adiponectin (46). All these changes increase the risk of atherosclerosis (46), through insulin resistance and the pro-inflammatory and the thrombotic state of visceral obesity (47). The portal hypothesis postulates that the hyper-lipolytic state of intra-abdominal VAT results in the release of non-esterified free fatty acids (46, 48), which through the portal circulation reaches the liver, resulting in increased hepatic glucose production, decreased insulin clearance and hyperinsulinemia, and thus increased very low dense lipoproteins -apolipoprotein B secretion and hypertriglyceridemia (47). VAT is also associated with a decrease in high density lipoprotein, which similarly becomes small and dense (46). This may contribute to hepatic pathology including non-alcoholic fatty liver disease (49). Another hypothesis states that visceral fat is a form of “ectopic fat” accumulation, a marker of energy imbalance and relative inability of subcutaneous adipose tissue to store body fat (47, 50). Therefore, the cardio-metabolic risk factors may be the mediators of the adverse impact of high abdominal VAT parameters. As demonstrated in several cohort studies included in our SR, the association of VAT and all-cause mortality lost significance after adjusting for metabolic disorders, including DM, high BMI, fatty liver and others, with few exceptions (30, 33).

Our findings, derived from observational data, suggest that age seems to have a significant effect modifier on the association of VAT and all-cause mortality, as it has been described previously for BMI. In older individuals (≥ 70 years), a higher BMI was protective against mortality (51, 52). In fact, while in younger individuals increased adiposity implies deleterious metabolic events, in older adults, this relationship becomes neutral or even reversed, secondary to reverse causality, confounders or other competing risks of mortality (53, 54). Several non-communicable diseases showed better survival in individuals with obesity, including end stage kidney disease, congestive heart failure, hypertension, and others (55). This survival resiliency that is conferred by adiposity may reflect better nutritional reserve during illnesses, improved wellbeing (28), in addition to better mitochondrial energetics (56). Noteworthy, the paradoxical findings of better survival with higher weight has been mostly based on BMI data, known to have several limitations, and was challenged by potential confounders, including an earlier detection of disease in individuals with obesity, compared to normal and underweight individuals (57). The lack of a detrimental effect of obesity might be a surrogate of worse health in individuals with normal weight (58), possibly related to unintentional weight loss secondary to cancer or other pro-inflammatory chronic diseases, frailty and higher rates of smoking in patients who are underweight (57). Another explanation could be the change in body composition, the loss of lean mass in parallel to the decrease in fat mass, resulting in increased oxidative stress and chronic inflammation (59), which may contribute to the worse outcome in older individuals who are lean (60), although this has not been widely investigated. Finally, the Health ABC and AGES-Reykjavik cohorts demonstrated that VAT density, inversely proportional to VAT area, is a predictor of mortality in older (66-96 years) individuals followed for 14 and 4-9 years, respectively (27). In fact, the groups with the highest VAT density lost weight during follow up, and this might have confounded the results (27). Such findings may suggest that advocating for weight loss in older adults should be cautiously done on an individual basis and accompanied with appropriate measures to preserve lean mass (12).

## Strength and Limitations

To our knowledge, this is the first SR on the relation between abdominal VAT and all-cause mortality. It mainly captured prospective observational studies from multiple databases with no time restriction. Major limitations of our review are related to the wide heterogeneity across various dimensions in the included studies: the population of interest, the imaging modality, and the definition of the intra-abdominal VAT borders. Three studies, using CT for VAT measurement, utilized the area of interest as umbilical level (28, 29, 32), whereas one study utilized the L1 level (34), six studies utilized the L4-L5 (10, 26, 27, 30, 31) or just above S1 (11) as the area of measurement. However, it has been shown that the inter-observer reproducibility of the measurement going through the umbilicus is consistent with that obtained using the L3-L4 disc (61). The analysis methods, the selected confounders, and the VAT area categories cutoff also differed widely across studies and rendered data pooling challenging. While previous literature suggested that a VAT area of 100 cm^2^ indicates an increased risk and 160 cm^2^ indicates a high risk for an adverse metabolic profile (62), the association of these specific cutoffs with mortality was not confirmed. Individual studies have explored various cutoffs, depending on the distribution of their data. The duration of follow up was 5-10 years for most studies (11, 26, 28, 29, 31, 33), with few extending over a shorter median follow up of 2-4 years (10, 33, 34) or more than 10 years (27, 32). We only identified observational studies, and despite adjusted analyses in individual studies, the effect of confounding cannot be completely excluded, and causality cannot be established in the absence of interventional studies.

## Implication and Gaps for Future Research

Increased VAT area seems to be associated with all-cause mortality, more so in younger individuals, mediated by metabolic parameters, BMI and glycemic indices. The association weakens in older individuals, most likely secondary to reverse causality. Further prospective studies using homogenous VAT measurement, categorization and analysis methods are needed. Ultimately, an Individual Participant Data meta-analysis would allow the identification of a specific abdominal VAT area cutoff associated with a high mortality risk, and the evaluation of the impact of specific predictors on such association.

## Data Availability Statement

The original contributions presented in the study are included in the article/**Supplementary Material**. Further inquiries can be directed to the corresponding author.

## Author Contributions

Design: MC, RS, and MG. Data collection: MC, RS, MG, and RH. Analysis: MC, RS, MG, and AK. Manuscript write-up: MC, RS, MG, RH, AK, KS, and JB. All authors contributed to the article and approved the submitted version.

## Conflict of Interest

KS: ownership and management interest in Intellihealth. JB: equity in SynchroHealth LLC.

The remaining authors declare that the research was conducted in the absence of any commercial or financial relationships that could be construed as a potential conflict of interest.

## Publisher’s Note

All claims expressed in this article are solely those of the authors and do not necessarily represent those of their affiliated organizations, or those of the publisher, the editors and the reviewers. Any product that may be evaluated in this article, or claim that may be made by its manufacturer, is not guaranteed or endorsed by the publisher.

## Abbreviations

BMI, body mass index; SAT, subcutaneous adipose tissue; VAT, visceral adipose tissue; CV,cardiovascular; CT, computed tomography; MRI, magnetic resonance imaging’; DXA dual-energy x-ray absorptiometry; SR, systematic review; US, United States; ROB, risk of bias; NOS, Newcastle-Ottawa Quality Assessment Scale; HR, hazard ratio; OR, odds ratio; MESA, Multi-Ethnic Study of Atherosclerosis; Health ABC, Health, Aging, and Body Composition; AGES-Reykjavik, Age, Gene and/or Environment Susceptibility-Reykjavik; DM, diabetes mellitus; KLoSHA, Korean Longitudinal study on Health and Aging; SD, standard deviation; IQR, interquartile range; TNF-α, tumor necrosis factor-α.
