# Supplementary material for: Abdominal Visceral Adipose Tissue and All-Cause Mortality: A Systematic Review
Source: Front Endocrinol (Lausanne). 2022 Aug 22;13:922931. doi: 10.3389/fendo.2022.922931 (PMC9446237; doi:10.3389/fendo.2022.922931)
Supplement: Supplementary file 1 [file DataSheet_1.docx]

**Appendix 1. PRISMA checklist**

| **Section/topic** | **#** | **Checklist item** | **Reported on page #** |
| --- | --- | --- | --- |
| **TITLE** | | |  |
| Title | 1 | Identify the report as a systematic review, meta-analysis, or both. | 1 |
| **ABSTRACT** | | |  |
| Structured summary | 2 | Provide a structured summary including, as applicable: background; objectives; data sources; study eligibility criteria, participants, and interventions; study appraisal and synthesis methods; results; limitations; conclusions and implications of key findings; systematic review registration number. | 3 |
| **INTRODUCTION** | | |  |
| Rationale | 3 | Describe the rationale for the review in the context of what is already known. | 4 |
| Objectives | 4 | Provide an explicit statement of questions being addressed with reference to participants, interventions, comparisons, outcomes, and study design (PICOS). | 4 |
| **METHODS** | | |  |
| Protocol and registration | 5 | Indicate if a review protocol exists, if and where it can be accessed (e.g., Web address), and, if available, provide registration information including registration number. | 4 |
| Eligibility criteria | 6 | Specify study characteristics (e.g., PICOS, length of follow-up) and report characteristics (e.g., years considered, language, publication status) used as criteria for eligibility, giving rationale. | 4-5 |
| Information sources | 7 | Describe all information sources (e.g., databases with dates of coverage, contact with study authors to identify additional studies) in the search and date last searched. | 4-5 |
| Search | 8 | Present full electronic search strategy for at least one database, including any limits used, such that it could be repeated. | Appendix 2a |
| Study selection | 9 | State the process for selecting studies (i.e., screening, eligibility, included in systematic review, and, if applicable, included in the meta-analysis). | 5 |
| Data collection process | 10 | Describe method of data extraction from reports (e.g., piloted forms, independently, in duplicate) and any processes for obtaining and confirming data from investigators. | 5-6 |
| Data items | 11 | List and define all variables for which data were sought (e.g., PICOS, funding sources) and any assumptions and simplifications made. | 5-6 |
| Risk of bias in individual studies | 12 | Describe methods used for assessing risk of bias of individual studies (including specification of whether this was done at the study or outcome level), and how this information is to be used in any data synthesis. | 6 |
| Summary measures | 13 | State the principal summary measures (e.g., risk ratio, difference in means). | 6 |
| Synthesis of results | 14 | Describe the methods of handling data and combining results of studies, if done, including measures of consistency (e.g., I^2^) for each meta-analysis. | 6 |
| Risk of bias across studies | 15 | Specify any assessment of risk of bias that may affect the cumulative evidence (e.g., publication bias, selective reporting within studies). | 6 |
| Additional analyses | 16 | Describe methods of additional analyses (e.g., sensitivity or subgroup analyses, meta-regression), if done, indicating which were pre-specified. | NA |
| **RESULTS** | | | |
| Study selection | 17 | Give numbers of studies screened, assessed for eligibility, and included in the review, with reasons for exclusions at each stage, ideally with a flow diagram. | 6,  Figure 1 flow diagram, Appendix 2b |
| Study characteristics | 18 | For each study, present characteristics for which data were extracted (e.g., study size, PICOS, follow-up period) and provide the citations. | 6-7, Table 1 |
| Risk of bias within studies | 19 | Present data on risk of bias of each study and, if available, any outcome level assessment (see item 12). | 7, Table 1, Appendix 4b |
| Results of individual studies | 20 | For all outcomes considered (benefits or harms), present, for each study: (a) simple summary data for each intervention group (b) effect estimates and confidence intervals, ideally with a forest plot. | NA |
| Synthesis of results | 21 | Present the main results of the review. If meta-analyses are done, include for each, confidence intervals and measures of consistency. | 10-13 |
| Risk of bias across studies | 22 | Present results of any assessment of risk of bias across studies (see Item 15). | Table 1, Appendix 4b |
| Additional analysis | 23 | Give results of additional analyses, if done (e.g., sensitivity or subgroup analyses, meta-regression [see Item 16]). | NA |
| **DISCUSSION** | | | |
| Summary of evidence | 24 | Summarize the main findings including the strength of evidence for each main outcome; consider their relevance to key groups (e.g., healthcare providers, users, and policy makers). | 13-15 |
| Limitations | 25 | Discuss limitations at study and outcome level (e.g., risk of bias), and at review-level (e.g., incomplete retrieval of identified research, reporting bias). | 15 |
| Conclusions | 26 | Provide a general interpretation of the results in the context of other evidence, and implications for future research. | 16 |
| **FUNDING** | | | |
| Funding | 27 | Describe sources of funding for the systematic review and other support (e.g., supply of data); role of funders for the systematic review. | NA |

**Appendix 2a. Search Strategy**

**Ovid MEDLINE(R) Epub Ahead of Print, In-Process & Other Non-Indexed Citations, Ovid MEDLINE(R) Daily and Ovid MEDLINE(R) <1946 to Present>**

| **Search** | **Query** |
| --- | --- |
| **Concept 1: VAT** | |
| **1** | obesity, abdominal/ |
| **2** | abdominal fat/ or intra-abdominal fat/ |
| **3** | ((abdom?n* or (intra adj abdom?n*) or viscera* or central or mesenter* or (retro adj periton*) or periton*) adj2 (obesit* or fat* or adipos* or tissue*)).mp. |
| **4** | exp Body Composition/ |
| **5** | (body adj4 (composition* or fat* or adipos* or anthropometr*)).mp. |
| **6** | adipose tissue/ or adipose tissue, white/ |
| **7** | (adiposity or ((adipose or white or fatty) adj2 tissue*)).mp. |
| **8** | (fat adj2 (bod* or pad? or tissue* or white)).mp. |
| **9** | 6 or 7 or 8 |
| **10** | abdomen/ or exp abdominal cavity/ |
| **11** | (abdom?n* or (intra adj abdom?n*) or viscera* or central or mesenter* or (retro adj periton*) or periton*).mp. |
| **12** | 10 or 11 |
| **13** | 9 AND 12 |
| **14** | 1 or 2 or 3 or 4 or 5 or 13 |
| **Concept 2: Imaging** | |
| **15** | magnetic resonance imaging/ or exp diffusion magnetic resonance imaging/ |
| **16** | (MRI* or NMR* or FMRI* or zeugmatogra*).mp. |
| **17** | (((chemical adj shift*) or (spin adj echo*) or (proton* adj spin) or (magneti* adj (transfer or resonance)) or (diffusion adj tensor*) or MR) adj4 (imag* or tomogra* or scan*)).mp. |
| **18** | (diffusion* adj2 tractogra*).mp. |
| **19** | tomography, x-ray computed/ or four-dimensional computed tomography/ or exp tomography, spiral computed/ |
| **20** | (((Electron adj beam*) or comput* or (x adj ray*)) adj4 tomogra*).mp. |
| **21** | ((CT? or cat) adj4 (scan* or (x adj ray*) or 4d or (four adj dimension*) or helical or spiral)).mp. |
| **22** | Tomodensitomet*.mp. |
| **23** | exp Densitometry/ |
| **24** | (Hologic or Lunar or Norland or densitomet* or (Photo adj densitomet*) or dexa or dxa).mp. |
| **25** | ((Absorptiomet* or scan* or imag*) adj4 (dpx or (dual adj energy) or photon* or (x adj ray*))).mp. |
| **26** | or/15-25 |
| **Concept 3: Outcome of all-cause mortality or cardiovascular outcomes** | |
| **27** | exp Myocardial Infarction/ |
| **28** | (((heart or myocardia* or cardia* or (cardio adj vascular) or coronar*) adj2 (attack* or infarct* or infract* or accident* or event* or outcome* or shock* or disease* or Isch?emia* or disorder* or thromb* or embol* or occlusion*)) or MI or nstemi* or stemi*).mp. |
| **29** | stroke/ or brain infarction/ or exp brain stem infarctions/ or cerebral infarction/ or stroke, lacunar/ |
| **30** | ((cereb* or brain or (intra adj cranial) or (brain adj stem*) or sub?cortic* or (choroidal adj arter*) or (heubner* adj arter*) or aca or mca or pca or lacunar) adj4 (infarct* or infract* or accident* or event* or disease* or Isch?emia* or disorder* or thromb* or embol* or occlu*)).mp. |
| **31** | (Apoplex* or stroke* or cva?).mp. |
| **32** | ((benedict or claude or foville or (millard adj gublar) or weber or medullary or (vieseaux adj wallenberg*) or wallenberg* or (top adj3 basilar) or (lateral adj bulbar) or (inferior adj cerebellar) or (cerebral adj arter*) or lacunar) adj2 syndrome*).mp. |
| **33** | exp Angina, Unstable/ |
| **34** | (Angina* adj2 (unstable or (pre adj (infarct* or infract*)) or (at adj rest) or pector* or prinzmetal*)).mp. |
| **35** | (myocardia* adj2 (pre adj (infarct* or infract*))).mp. |
| **36** | Cerebrovascular Disorders/ |
| **37** | mortality.fs. (566528) |
| **38** | (death* or mortalit* or fatal*).mp. |
| **39** | Death/ or mortality/ or fatal outcome/ |
| **40** | or/27-39 |
| **Concept 1 + 2+ 3 combined** | |
| **41** | 14 and 26 and 40 |

**Cochrane Library search strategy**

| **Search** | **Query** |
| --- | --- |
| **Concept 1: VAT** | |
| #1 | MeSH descriptor: [Obesity, Abdominal] this term only |
| #2 | MeSH descriptor: [Abdominal Fat] this term only |
| #3 | MeSH descriptor: [Intra-Abdominal Fat] this term only |
| #4 | ((abdom?n* OR (intra NEXT abdom?n*) OR intraabdom?n* OR viscera* OR central OR mesenter* OR (retro NEXT periton*) OR retroperiton* OR periton*) NEAR/2 (obesit* OR fat* OR adipos* OR tissue*)) |
| #5 | MeSH descriptor: [Body Composition] explode all trees |
| #6 | (body NEAR/4 (composition* OR fat* OR adipos* OR anthropometr*)) |
| #7 | MeSH descriptor: [Adipose Tissue] this term only |
| #8 | MeSH descriptor: [Adipose Tissue, White] this term only |
| #9 | (adiposity OR ((adipose OR white OR fatty) NEAR/2 tissue*)) |
| #10 | (fat NEAR/2 (bod* OR pad? OR tissue* OR white)) |
| #11 |  |
| #12 | MeSH descriptor: [Abdomen] this term only |
| #13 | MeSH descriptor: [Abdominal Cavity] explode all trees |
| #14 | (abdom?n* OR (intra NEXT abdom?n*) OR intraabdom?n* OR viscera* OR central OR mesenter* OR (retro NEXT periton*) OR retroperiton* OR periton*) |
| #15 |  |
| #16 | #11 AND #15 |
| #17 | OR #16 |
| **Concept 2: Imaging** | |
| #18 | MeSH descriptor: [Magnetic Resonance Imaging] this term only |
| #19 | MeSH descriptor: [Diffusion Magnetic Resonance Imaging] explode all trees |
| #20 | (MRI* OR NMR* OR FMRI* OR zeugmatogra*) |
| #21 | (((chemical NEXT shift*) OR (spin NEXT echo*) OR (proton* NEXT spin) OR (magneti* NEXT (transfer OR resonance)) OR (diffusion NEXT tensor*) or MR) NEAR/4 (imag* OR tomogra* OR scan*)) |
| #22 | (diffusion* NEAR/2 tractogra*) |
| #23 | MeSH descriptor: [Tomography, X-Ray Computed] this term only |
| #24 | MeSH descriptor: [Four-Dimensional Computed Tomography] this term only |
| #25 | MeSH descriptor: [Tomography, Spiral Computed] explode all trees |
| #26 | (((Electron NEXT beam*) OR comput* OR (x NEXT ray*) OR xray*) NEAR/4 tomogra*) |
| #27 | ((CT? OR cat) NEAR/4 (scan* OR (x NEXT ray*) OR xray* OR 4d OR (four NEXT dimension*) OR helical OR spiral)) |
| #28 | Tomodensitomet* |
| #29 | MeSH descriptor: [Densitometry] explode all trees |
| #30 | (Hologic OR Lunar OR Norland OR densitomet* OR (Photo NEXT densitomet*) OR photodensitomet* OR dexa OR dxa) |
| #31 | ((Absorptiomet* OR scan* OR imag*) NEAR/4 (dpx OR (dual NEXT energy) OR photon* OR (x NEXT ray*) OR xray*)) |
| #32 | {OR #18-#31} |
| **Concept 3: Outcome of all-cause mortality or cardiovascular outcomes** | |
| #33 | MeSH descriptor: [Myocardial Infarction] explode all trees |
| #34 | (((heart OR myocardia* OR cardia* OR (cardio NEXT vascular) OR coronar*) NEAR/2 (attack* OR infarct* OR infract* OR accident* OR event* OR outcome* OR shock* OR disease* OR Isch?emia* OR disorder* OR thromb* OR embol* OR occlusion*)) OR MI OR nstemi* OR stemi*) |
| #35 | MeSH descriptor: [Stroke] this term only |
| #36 | MeSH descriptor: [Brain Infarction] this term only |
| #37 | MeSH descriptor: [Brain Stem Infarctions] explode all trees |
| #38 | MeSH descriptor: [Cerebral Infarction] this term only |
| #39 | MeSH descriptor: [Stroke, Lacunar] this term only |
| #40 | ((cereb* OR brain OR (intra NEXT cranial) OR intracranial OR (brain NEXT stem*) OR brainstem* OR sub?cortic* OR (choroidal NEXT arter*) OR (heubner* NEXT arter*) OR aca OR mca OR pca OR lacunar) NEAR/4 (infarct* OR infract* OR accident* OR event* OR disease* OR Isch?emia* OR disorder* OR thromb* OR embol* OR occlu*)) |
| #41 | (Apoplex* OR stroke* OR cva?) |
| #42 | ((benedict OR claude OR foville OR (millard NEXT gublar) OR weber OR medullary OR (vieseaux NEXT wallenberg*) OR wallenberg* OR (top NEXT/3 basilar) OR (lateral NEXT bulbar) OR (inferior NEXT cerebellar) OR (cerebral NEXT arter*) OR lacunar) NEAR/2 syndrome*) |
| #43 | MeSH descriptor: [Angina, Unstable] explode all trees |
| #44 | (Angina* NEAR/2 (unstable OR (pre NEXT (infarct* OR infract*)) OR (at NEXTj rest) OR pector* OR prinzmetal*)) |
| #45 | (myocardia* NEAR/2 (pre NEXT (infarct* OR infract*))) |
| #46 | MeSH descriptor: [Cerebrovascular Disorders] this term only |
| #47 | (death* OR mortalit* OR fatal*) |
| #48 | MeSH descriptor: [Death] this term only |
| #49 | MeSH descriptor: [Mortality] this term only |
| #50 | MeSH descriptor: [Fatal Outcome] this term only |
| #51 |  |
| **Concept 1 + 2+ 3 combined** | |
| #52 | #17 AND #32 AND #51 |

**Embase search strategy**

| **Search** | **Query** |
| --- | --- |
| **Concept 1: VAT** | |
| #1. | 'abdominal obesity'/de OR 'abdominal fat'/de OR 'intra-abdominal fat'/exp OR 'mesenteric fat'/de OR 'central obesity'/de OR 'visceral obesity'/de |
| #2. | ((abdom?n* OR intraabdom?n* OR 'intra abdom?n' OR viscera* OR central OR mesenter* OR retroperiton* OR 'retro periton*') NEAR/2 (obesit* OR fat* OR adipos* OR tissue*)):ti,ab,kw |
| #3. | 'adipose tissue'/de OR 'body fat'/de OR 'fat pad'/de OR 'white adipose tissue'/de OR 'white adipocyte'/de |
| #4. | adiposity:ti,ab,kw OR (((adipose OR white OR fatty) NEAR/2 tissue*):ti,ab,kw |
| #5. | (fat NEAR/2 (bod* OR pad$ OR tissue* OR white)):ti,ab,kw |
| #6. | #3 OR #4 OR #5 |
| #7. | 'abdomen'/de OR 'abdominal cavity'/de OR 'abdominal viscera'/de OR 'peritoneum'/de OR 'mesentery'/de OR 'retroperitoneum'/de |
| #8. | abdom?n*:ti,ab,kw OR intraabdom?n*:ti,ab,kw OR 'intra abdom?n':ti,ab,kw OR viscera*:ti,ab,kw OR central:ti,ab,kw OR mesenter*:ti,ab,kw OR retroperiton*:ti,ab,kw OR 'retro periton*':ti,ab,kw |
| #9. | #7 OR #8 |
| #10. | #6 AND #9 |
| #11. | 'body composition'/de OR 'body distribution'/de OR 'body fat distribution'/de |
| #12. | ((composition* OR anthropometr* OR fat* OR adipos*) NEAR/4 body):ti,ab,kw |
| #13. | #1 OR #2 OR #10 OR #11 OR #12 |
| **Concept 2: Imaging** | |
| #14. | 'nuclear magnetic resonance imaging'/de OR 'diffusion tensor imaging'/de OR 'diffusion weighted imaging'/de OR 'dynamic contrast-enhanced magnetic resonance imaging'/de OR 'echo planar imaging'/de OR 'fluorine magnetic resonance imaging'/de OR 'functional magnetic resonance imaging'/exp OR 'perfusion weighted imaging'/de OR 'whole body mri'/de OR 'computer assisted tomography'/de OR 'computer assisted impedance tomography'/de OR 'cone beam computed tomography'/de OR 'electron beam tomography'/de OR 'four dimensional computed tomography'/de OR 'high resolution computer tomography'/de OR 'micro-computed tomography'/de OR 'multidetector computed tomography'/de OR 'optical tomography'/de OR 'photoacoustic tomography'/de OR 'spiral computer assisted tomography'/de OR 'thermoacoustic tomography'/de OR 'whole body ct'/de OR 'x-ray computed tomography'/exp OR 'computer assisted radiography'/de OR 'whole body tomography'/de |
| #15. | mri$:ti,ab,kw OR nmr$:ti,ab,kw OR fmri$:ti,ab,kw OR zeugmatogra*:ti,ab,kw |
| #16. | (('chemical shift*' OR 'spin echo*' OR 'echo* plannar' OR 'proton$ spin' OR 'magneti* transfer' OR 'magneti* resonance' OR 'diffusion tensor$' OR 'diffusion weighted' OR 'perfusion weighted' OR mr) NEAR/4 (imag* OR tomogra* OR scan*)):ti,ab,kw |
| #17. | (diffusion* NEAR/2 tractogra*):ti,ab,kw |
| #18. | (('electron beam*' OR comput* OR 'x ray$' OR xray$) NEAR/4 tomogra*):ti,ab,kw |
| #19. | ((ct$ OR cat) NEAR/4 (scan* OR 'x ray$' OR xray$ OR 4d OR 'four dimension*' OR helical OR spiral)):ti,ab,kw |
| #20. | tomodensitomet*:ti,ab,kw |
| #21. | 'dual energy x ray absorptiometry'/de OR 'x ray absorptiometry'/de OR 'absorptiometry'/de OR 'densitometry'/exp |
| #22. | hologic:ti,ab,kw OR lunar:ti,ab,kw OR norland:ti,ab,kw OR densitomet*:ti,ab,kw OR 'photo densitomet*':ti,ab,kw OR photodensitomet*:ti,ab,kw OR dexa:ti,ab,kw OR dxa:ti,ab,kw |
| #23. | ((absorptiomet* OR scan* OR imag*) NEAR/4 (dpx OR 'dual energy' OR photon* OR 'x ray$' OR xray$)):ti,ab,kw |
| #24. | #14 OR #15 OR #16 OR #17 OR #18 OR #19 OR #20 OR #21 OR #22 OR #23 |
| **Concept 3: Outcome of all-cause mortality or cardiovascular outcomes** | |
| #25 | 'mortality'/de OR 'cardiovascular mortality'/de OR 'death'/de OR 'fatality'/de OR mortality:lnk |
| #26. | death*:ti,ab,kw OR mortalit*:ti,ab,kw OR fatal*:ti,ab,kw |
| #27. | 'heart ventricle infarction'/exp OR 'heart muscle necrosis'/de OR 'heart infarction size'/de OR 'heart atrium infarction'/de OR 'anterior myocardial infarction'/de OR 'inferior myocardial infarction'/de OR 'non st segment elevation myocardial infarction'/de OR 'posterior myocardial infarction'/de OR 'silent myocardial infarction'/de OR 'st segment elevation myocardial infarction'/de OR 'heart infarction'/de |
| #28. | (((heart OR myocardia* OR cardia OR 'cardio vascular' OR coronar*) NEAR/2 (necrosis OR attack* OR infarct* OR infract* OR accident* OR event* OR outcome* OR shock* OR disease* OR isch$emia* OR disorder* OR thromb* OR embol* OR occlusion*)):ti,ab,kw) OR mi:ti,ab,kw OR nstemi*:ti,ab,kw OR stemi*:ti,ab,kw |
| #29. | 'brain infarction'/exp OR 'brain ischemia'/de OR 'transient ischemic attack'/de OR 'cerebrovascular accident'/de OR 'cardioembolic stroke'/de OR 'lacunar stroke'/de OR 'basilar artery obstruction'/de OR 'middle cerebral artery occlusion'/de OR 'occlusive cerebrovascular disease'/de OR 'wallenberg syndrome'/de OR 'cerebral artery disease'/de |
| #30. | ((cereb* OR brain OR 'intra cranial' OR intracranial OR 'brain stem$' OR brainstem$ OR sub$cortic* OR 'choroidal arter*' OR 'heubner* arter*' OR aca OR mca OR pca OR lacunar) NEAR/4 (infarct* OR infract* OR accident* OR event* OR disease* OR isch$emia* OR disorder* OR thromb* OR embol* OR occlu*)):ti,ab,kw |
| #31. | apoplex*:ti,ab,kw OR stroke*:ti,ab,kw OR cva$:ti,ab,kw |
| #32. | ((benedict OR claude OR foville OR 'millard gublar' OR millardgublar OR weber OR medullary OR 'vieseaux wallenberg*' OR wallenberg* OR 'top basilar' OR 'lateral bulbar' OR 'inferior cerebellar' OR 'cerebral arter*' OR lacunar) NEAR/2 syndrome*):ti,ab,kw |
| #33. | 'unstable angina pectoris'/exp |
| #34. | (angina* NEAR/2 (unstable OR 'pre infarct*' OR preinfarct* OR 'pre infract*' OR preinfract* OR 'at rest' OR pector* OR prinzmetal*)):ti,ab,kw |
| #35. | (myocardia* NEAR/2 ('pre infarct*' OR preinfarct*OR 'pre infract*' OR preinfract*)):ti,ab,kw |
| #36. | #25 OR #26 OR #27 OR #28 OR #29 OR #30 OR #31 OR #32 OR #33 OR #34 OR #35 |
| **Concept 1 + 2+ 3 combined** | |
| #37. | #13 AND #24 AND #36 |

Cinahl Library

| **Search** | **Query** |
| --- | --- |
| **Concept 1: VAT** | |

| S1: | (MH "Abdominal Fat") |
| --- | --- |
| S2: | TI ((abdom#n* OR (intra W0 abdom#n*) OR intraabdom#n* OR viscera* OR central OR mesenter* OR (retro W0 periton*) OR retroperiton* OR periton*) N2 (obesit* OR fat* OR adipos* OR tissue*)) OR AB ((abdom#n* OR (intra W0 abdom#n*) OR intraabdom#n* OR viscera* OR central OR mesenter* OR (retro W0 periton*) OR retroperiton* OR periton*) N2 (obesit* OR fat* OR adipos* OR tissue*)) OR MW ((abdom#n* OR (intra W0 abdom#n*) OR intraabdom#n* OR viscera* OR central OR mesenter* OR (retro W0 periton*) OR retroperiton* OR periton*) N2 (obesit* OR fat* OR adipos* OR tissue*)) |
| S3: | (MH "Body Composition+") |
| S4: | TI (body N4 (composition* OR fat* OR adipos* OR anthropometr*)) OR AB (body N4 (composition* OR fat* OR adipos* OR anthropometr*)) OR MW (body N4 (composition* OR fat* OR adipos* OR anthropometr*)) |
| S5: | (MH "Adipose Tissue") |
| S6: | TI (adiposity OR ((adipose OR white OR fatty) N2 tissue*)) OR AB (adiposity OR ((adipose OR white OR fatty) N2 tissue*)) OR MW (adiposity OR ((adipose OR white OR fatty) N2 tissue*)) |
| S7: | TI (fat N2 (bod* OR pad# OR tissue* OR white)) OR AB (fat N2 (bod* OR pad# OR tissue* OR white)) OR MW (fat N2 (bod* OR pad# OR tissue* OR white)) |
| S8: | S5 OR S6 OR S7 |
| S9: ( | (MH "Peritoneum+") OR (MH "Retroperitoneal Space") OR (MH "Abdomen") |
| S10: | TI (abdom#n* OR (intra W0 abdom#n*) OR intraabdom#n* OR viscera* OR central OR mesenter* OR (retro w0 periton*) OR retroperiton* or periton*) OR AB (abdom#n* OR (intra w0 abdom#n*) OR intraabdom#n* OR viscera* OR central OR mesenter* OR (retro W0 periton*) OR retroperiton* OR periton*) OR MW (abdom#n* OR (intra W0 abdom#n*) OR intraabdom#n* OR viscera* OR central OR mesenter* OR (retro W0 periton*) OR retroperiton* OR periton*) |
| S11: | S9 OR S10 |
| S12: | S8 AND S11 |
| S13: | S1 OR S2 OR S3 OR S4 OR S12 |
| **Concept 2: Imaging** | |
| S14: | (MH "Magnetic Resonance Imaging") |
| S15: | TI (MRI* OR NMR* OR FMRI* OR zeugmatogra*) OR AB (MRI* OR NMR* OR FMRI* OR zeugmatogra*) OR MW (MRI* OR NMR* OR FMRI* OR zeugmatogra*) |
| S16: | TI (((chemical W0 shift*) OR (spin W0 echo*) OR (proton* W0 spin) OR (magneti* W0 (transfer OR resonance)) OR (diffusion W0 tensor*) or MR) N4 (imag* or tomogra* or scan*)) OR AB (((chemical W0 shift*) OR (spin W0 echo*) OR (proton* W0 spin) OR (magneti* W0 (transfer OR resonance)) OR (diffusion W0 tensor*) or MR) N4 (imag* or tomogra* or scan*)) OR MW(((chemical W0 shift*) OR (spin W0 echo*) OR (proton* W0 spin) OR (magneti* W0 (transfer OR resonance)) OR (diffusion W0 tensor*) or MR) N4 (imag* or tomogra* or scan*)) |
| S17: | TI (diffusion* N2 tractogra*) OR AB (diffusion* N2 tractogra*) OR MW (diffusion* N2 tractogra*) |
| S18: | (MH "Tomography, X-Ray Computed") OR (MH "Tomography, Spiral Computed+") |
| S19: | TI (((Electron W0 beam*) OR comput* OR (x W0 ray*) OR xray*) N4 tomogra*) OR AB (((Electron W0 beam*) OR comput* OR (x W0 ray*) OR xray*) N4 tomogra*) OR MW (((Electron W0 beam*) OR comput* OR (x W0 ray*) OR xray*) N4 tomogra*) |
| S20: | TI ((CT# OR cat) N4 (scan* OR (x W0 ray*) OR xray* OR 4d OR (four W0 dimension*) OR helical OR spiral)) OR AB ((CT# OR cat) N4 (scan* OR (x W0 ray*) OR xray* OR 4d OR (four W0 dimension*) OR helical OR spiral)) OR MW ((CT# OR cat) N4 (scan* OR (x W0 ray*) OR xray* OR 4d OR (four W0 dimension*) OR helical OR spiral)) |
| S21: | TI Tomodensitomet* OR AB Tomodensitomet* OR MW Tomodensitomet* |
| S22: | (MH "Densitometry+") |
| S23: | TI (Hologic OR Lunar OR Norland OR densitomet* OR (Photo W0 densitomet*) OR Photodensitomet* OR dexa or dxa) OR AB (Hologic OR Lunar OR Norland OR densitomet* OR (Photo W0 densitomet*) OR Photodensitomet* OR dexa or dxa) OR MW (Hologic OR Lunar OR Norland OR densitomet* OR (Photo W0 densitomet*) OR Photodensitomet* OR dexa or dxa) |
| S24: | TI ((Absorptiomet* OR scan* OR imag*) N4 (dpx OR (dual W0 energy) OR dualenergy OR photon* OR (x W0 ray*) OR xray*)) OR AB ((Absorptiomet* OR scan* OR imag*) N4 (dpx OR (dual W0 energy) OR dualenergy OR photon* OR (x W0 ray*) OR xray*)) OR MW ((Absorptiomet* OR scan* OR imag*) N4 (dpx OR (dual W0 energy) OR dualenergy OR photon* OR (x W0 ray*) OR xray*)) |
| S25: | S14 OR S15 OR S16 OR S17 OR S18 OR S19 OR S20 OR S21 OR S22 OR S23 OR S24 |
| **Concept 3: Outcome of all-cause mortality or cardiovascular outcomes** | |
| S26: | (MH "Myocardial Infarction") |
| S27: | TI (((heart OR myocardia* OR cardia* OR (cardio W0 vascular) OR cardiovascular OR coronar*) N2 (attack* OR infarct* OR infract* OR accident* OR event* OR outcome* OR shock* OR disease* OR Isch#emia* OR disorder* OR thromb* OR embol* OR occlusion*)) OR MI OR nstemi* OR stemi*) OR AB(((heart OR myocardia* OR cardia* OR (cardio W0 vascular) OR cardiovascular OR coronar*) N2 (attack* OR infarct* OR infract* OR accident* OR event* OR outcome* OR shock* OR disease* OR Isch#emia* OR disorder* OR thromb* OR embol* OR occlusion*)) OR MI OR nstemi* OR stemi*) OR MW (((heart OR myocardia* OR cardia* OR (cardio W0 vascular) OR cardiovascular OR coronar*) N2 (attack* OR infarct* OR infract* OR accident* OR event* OR outcome* OR shock* OR disease* OR Isch#emia* OR disorder* OR thromb* OR embol* OR occlusion*)) OR MI OR nstemi* OR stemi*) |
| S28: | (MH "Stroke+") |
| S29: | TI ((cereb* OR brain OR (intra W0 cranial) OR intracranial OR (brain stem*) OR brainstem OR subcortic* OR (sub W0 cortic*) OR (choroidal W0 arter*) OR (heubner* W0 arter*) OR aca OR mca OR pca OR lacunar) N4 (infarct* OR infract* OR accident* OR event* OR disease* OR Isch#emia* OR disorder* OR thromb* OR embol* OR occlu*)) OR AB ((cereb* OR brain OR (intra W0 cranial) OR intracranial OR (brain stem*) OR brainstem OR subcortic* OR (sub W0 cortic*) OR (choroidal W0 arter*) OR (heubner* W0 arter*) OR aca OR mca OR pca OR lacunar) N4 (infarct* OR infract* OR accident* OR event* OR disease* OR Isch#emia* OR disorder* OR thromb* OR embol* OR occlu*)) OR MW ((cereb* OR brain OR (intra W0 cranial) OR intracranial OR (brain stem*) OR brainstem OR subcortic* OR (sub W0 cortic*) OR (choroidal W0 arter*) OR (heubner* W0 arter*) OR aca OR mca OR pca OR lacunar) N4 (infarct* OR infract* OR accident* OR event* OR disease* OR Isch#emia* OR disorder* OR thromb* OR embol* OR occlu*)) |
| S30: | TI (Apoplex* OR stroke* OR cva OR cvas) OR AB (Apoplex* OR stroke* OR cva OR cvas) OR MW (Apoplex* OR stroke* OR cva OR cvas) |
| S31: | TI ((benedict OR claude OR foville OR (millard W0 gublar) OR weber OR medullary OR (vieseaux W0 wallenberg*) OR wallenberg* OR (top W3 basilar) OR (lateral W0 bulbar) OR (inferior W0 cerebellar) OR (cerebral W0 arter*) OR lacunar) N2 syndrome*) OR AB ((benedict OR claude OR foville OR (millard W0 gublar) OR weber OR medullary OR (vieseaux W0 wallenberg*) OR wallenberg* OR (top W3 basilar) OR (lateral W0 bulbar) OR (inferior W0 cerebellar) OR (cerebral W0 arter*) OR lacunar) N2 syndrome*) OR MW ((benedict OR claude OR foville OR (millard W0 gublar) OR weber OR medullary OR (vieseaux W0 wallenberg*) OR wallenberg* OR (top W3 basilar) OR (lateral W0 bulbar) OR (inferior W0 cerebellar) OR (cerebral W0 arter*) OR lacunar) N2 syndrome*) |
| S32: | (MH "Angina, Unstable") |
| S33: | TI (Angina* N2 (unstable OR (un W0 stable) OR (pre W0 infarct*) OR preinfarct* OR (pre W0 infract*) OR preinfract* OR (at W0 rest) OR pector* OR prinzmetal*)) OR AB (Angina* N2 (unstable OR (un W0 stable) OR (pre W0 infarct*) OR preinfarct* OR (pre W0 infract*) OR preinfract* OR (at W0 rest) OR pector* OR prinzmetal*)) OR MW (Angina* N2 (unstable OR (un W0 stable) OR (pre W0 infarct*) OR preinfarct* OR (pre W0 infract*) OR preinfract* OR (at W0 rest) OR pector* OR prinzmetal*)) |
| S34: | TI (myocardia* N2 ((pre W0 infarct*) OR preinfarct* OR (pre W0 infract*) OR preinfract*)) OR AB (myocardia* N2 ((pre W0 infarct*) OR preinfarct* OR (pre W0 infract*) OR preinfract*)) OR MW (myocardia* N2 ((pre W0 infarct*) OR preinfarct* OR (pre W0 infract*) OR preinfract*)) |
| S35: | TI (death* OR mortalit* OR fatal*) OR AB (death* OR mortalit* OR fatal*) OR MW (death* OR mortalit* OR fatal*) |
| S36: | (MH "Cerebrovascular Disorders") |
| S37: | (MH "Death") |
| S38: | (MH "Mortality") |
| S39: | (MH "Fatal Outcome") |
| S40: | S26 OR S27 OR S28 OR S29 OR S30 OR S31 OR S32 OR S33 OR S34 OR S35 OR S36 OR S37 OR S38 OR S39 |
| **Concept 1 + 2+ 3 combined** | |
| S41: | S13 AND S25 AND S40 |

**Appendix 2b. List of excluded studies after full text screening, with the respective reason for exclusion**

| **Author** | **Title** | **Reason for Exclusion**  **(Codes)*** |
| --- | --- | --- |
| Abbasi, S.A. | Visceral adiposity and left ventricular remodeling: The Multi-Ethnic Study of Atherosclerosis | Code 2 |
| Abdulnour, J. | The effect of the menopausal transition on body composition and cardiometabolic risk factors: A Montreal-Ottawa New Emerging Team group study | Code 1 |
| AbouAssi, H. | Adipose depots, not disease-related factors, account for skeletal muscle insulin sensitivity in established and treated rheumatoid arthritis | Code 2 |
| Abraham, T.M. | Association between visceral and subcutaneous adipose depots and incident cardiovascular disease risk factors | Code 1 |
| Agha, G. | Adiposity is associated with DNA methylation profile in adipose tissue | Code 2 |
| Ahn, S.G. | Relationship of epicardial adipose tissue by echocardiography to coronary artery disease | Code 2 |
| Al-Attar, A. | Human body composition and immunity: Visceral adipose tissue produces IL-15 and muscle strength inversely correlates with NK Cell function in elderly humans | Code 1 |
| Alman, A.C. | Higher pericardial adiposity is associated with prevalent diabetes: The Coronary Artery Risk Development in Young Adults study | Code 2 |
| Alvarez, G.E. | Sympathetic neural activation in visceral obesity | Code 2 |
| Alvey, N.J. | Association of fat density with subclinical atherosclerosis | Code 2 |
| Anan, F. | Diabetic retinopathy is associated with visceral fat accumulation in Japanese type 2 diabetes mellitus patients | Code 2 |
| Anan, F. | Visceral fat accumulation is a significant risk factor for white matter lesions in Japanese type 2 diabetic patients | Code 2 |
| Anderson, A.L. | Dietary Patterns and Survival of Older Adults | Code 3 |
| Anderson, P. J. | Visceral fat and cardiovascular risk factors in Chinese NIDDM patients | Code 4 |
| Andrews, A.M. | Core Temperature in Service Members With and Without Traumatic Amputations During a Prolonged Endurance Event | Code 3 |
| Antony, B. | Body fat predicts an increase and limb muscle strength predicts a decrease in leptin in older adults over 2.6 years | Code 1 |
| Arad, Y. | Association of multiple risk factors and insulin resistance with increased prevalence of asymptomatic coronary artery disease by an electron-beam computed tomographic study | Code 2 |
| Araneta, M.R.G. | Subclinical coronary atherosclerosis in asymptomatic Filipino and white women | Code 1 |
| Arcaro, G. | Body fat distribution predicts the degree of endothelial dysfunction in uncomplicated obesity | Code 2 |
| Aubertin-Leheudre, M. | Isoflavones and clinical cardiovascular risk factors in obese postmenopausal women: a randomized double-blind placebo-controlled trial | Code 4 |
| Aubertin-Leheudre, M. | Effect of 6 months of exercise and isoflavone supplementation on clinical cardiovascular risk factors in obese postmenopausal women: a randomized, double-blind study | Code 4 |
| Auyeung, T.W. | Adiposity to muscle ratio predicts incident physical limitation in a cohort of 3,153 older adults--an alternative measurement of sarcopenia and sarcopenic obesity | Code 1 |
| Auyeung, T.W. | Survival in older men may benefit from being slightly overweight and centrally obese--a 5-year follow-up study in 4,000 older adults using DXA | Code 2 |
| Azrad, M. | Intra-abdominal adipose tissue is independently associated with sex-hormone binding globulin in premenopausal women | Code 4 |
| B. A. Derstine | Healthy US population reference values for CT visceral fat measurements and the impact of IV contrast, HU range, and spinal levels | Code 2 |
| B. K. Ballenger | Vascular hemodynamics and blood pressure differences between young and older women | Code 3 |
| B. Larsen | Muscle area and density and risk of all-cause mortality: The Multi-Ethnic Study of Atherosclerosis | Code 3 |
| Baker, J.F. | The Adiponectin Paradox in the Elderly: Associations With Body Composition, Physical Functioning, and Mortality | Code 3 |
| Bamberg, F. | Subclinical disease burden as assessed by whole-body MRI in subjects with prediabetes, subjects with diabetes, and normal control subjects from the general population: The KORA-MRI study | Code 2 |
| Banerji, M.A. | Body composition, visceral fat, leptin, and insulin resistance in Asian Indian men | Code 1 |
| Baragetti, A. | Subclinical atherosclerosis is associated with Epicardial Fat Thickness and hepatic steatosis in the general population | Code 2 |
| Barbosa-Yañez, R.L. | Acute endothelial benefits of fat restriction over carbohydrate restriction in type 2 diabetes mellitus: Beyond carbs and fats | Code 4 |
| Basu, A. | Freeze-dried strawberries lower serum cholesterol and lipid peroxidation in adults with abdominal adiposity and elevated serum lipids | Code 4 |
| Bebenek, M. | Effect of exercise and Cimicifuga racemosa (CR BNO 1055) on bone mineral density, 10-year coronary heart disease risk, and menopausal complaints: the randomized controlled Training and Cimicifuga racemosa Erlangen (TRACE) study | Code 2 |
| Beller, E. | Hepatic fat is superior to BMI, visceral and pancreatic fat as a potential risk biomarker for neurodegenerative disease | Code 2 |
| Benberin, V.V. | Metabolically healthy and unhealthy obesity in the Kazakh | Code 3 |
| Berentzen, T.L. | Waist circumference adjusted for body mass index and intra-abdominal fat mass | Code 1 |
| Bergman, F. | Increasing physical activity in office workers--the Inphact Treadmill study; a study protocol for a 13-month randomized controlled trial of treadmill workstations | Code 3 |
| Berker, D. | Compatibility of different methods for the measurement of visceral fat in different body mass index strata | Code 2 |
| Berryman, C.E. | Effects of daily almond consumption on cardiometabolic risk and abdominal adiposity in healthy adults with elevated LDL-cholesterol: a randomized controlled trial | Code 2 |
| Bettencourt, N. | Epicardial adipose tissue is an independent predictor of coronary atherosclerotic burden | Code 1 |
| Bettencourt, N. | Predictors of circulating endothelial progenitor cell levels in patients without known coronary artery disease referred for multidetector computed tomography coronary angiography | Code 1 |
| Bites, A.C. | Association between functional measures and mortality in older persons | Code 3 |
| Bolinder, J. | Effects of dapagliflozin on body weight, total fat mass, and regional adipose tissue distribution in patients with type 2 diabetes mellitus with inadequate glycemic control on metformin | Code 2 |
| Borel, A.L. | Sleep apnoea attenuates the effects of a lifestyle intervention programme in men with visceral obesity | Code 2 |
| Bouchi, R. | Luseogliflozin reduces epicardial fat accumulation in patients with type 2 diabetes: A pilot study | Code 2 |
| Boulé, N.G. | Physical fitness and the metabolic syndrome in adults from the Quebec Family Study | Code 1 |
| Braillon, P. | Precision of body composition measurements by dual energy X-ray absorptiometry | Code 2 |
| Brandon, L.J. | Do the Same Central Anthropometric Variables that Best Predict Blood Pressure in European Americans also Best Predict Blood Pressure in African Americans? | Code 1 |
| Bray, G.A. | A 9-mo randomized clinical trial comparing fat-substituted and fat-reduced diets in healthy obese men: the Ole Study | Code 4 |
| Bredella, M.A. | Effects of GH on body composition and cardiovascular risk markers in young men with abdominal obesity | Code 2 |
| Breuil, V. | Immune changes in post-menopausal osteoporosis: The Immunos study | Code 1 |
| Brinkley, T.E. | Hepatic lipase gene -514C>T variant is associated with exercise training-induced changes in VLDL and HDL by lipoprotein lipase | Code 1 |
| Brinkley, T.E. | Periaortic fat and cardiovascular risk: A comparison of high-risk older adults and age-matched healthy controls | Code 2 |
| Briot, K. | Abdominal adipose tissue predicts major cardiovascular events in systemic necrotising vasculitides | Code 5 |
| Brittain, E.L. | Echocardiographic Pulmonary Artery Systolic Pressure in the Coronary Artery Risk Development in Young Adults (CARDIA) Study: Associations With Race and Metabolic Dysregulation | Code 2 |
| Brown, A.J.M. | Does dapagliflozin regress left ventricular hypertrophy in patients with type 2 diabetes? A prospective, double-blind, randomised, placebo-controlled study | Code 2 |
| Brown, R.C. | Effects of high fat versus high carbohydrate diets on plasma lipids and lipoproteins in endurance athletes | Code 3 |
| Buford, T.W. | Multi-modal intervention to reduce cardiovascular risk among hypertensive older adults: Design of a randomized clinical trial | Code 3 |
| Bunck, M.C. | Exenatide affects circulating cardiovascular risk biomarkers independently of changes in body composition | Code 2 |
| Burkhard, L. | Active Depression is Associated with Regional Adiposity in the Upper Abdomen and the Neck | Code 1 |
| C. Cui | Associations of body composition with incident dementia in older adults: Cardiovascular Health Study-Cognition Study | Code 4 |
| C. Diaz‐Canestro | Sex dimorphism in cardiac and aerobic capacities: The influence of body composition | Code 2 |
| C. Guo-Chong | Body Fat Distribution, Cardiometabolic Traits, and Risk of Major Lower-Extremity Arterial Disease in Postmenopausal Women | Code 4 |
| C. W. Puleo | Factors associated with baseline and serial changes in circulating NT-proBNP and high-sensitivity cardiac troponin T in a population-based cohort (Dallas Heart Study) | Code 1 |
| C. Zheng | Visceral Adipose Tissue Indices Independently Correlated with Obstructive Sleep Apnea in Patients with Type 2 Diabetes | Code 2 |
| Calvani, R. | Application of NMR-based Metabolomics to the Study of Gut Microbiota in Obesity | Code 2 |
| Cardoso-Saldaña, G.C. | Fatty liver and abdominal fat relationships with high C-reactive protein in adults without coronary heart disease | Code 2 |
| Carey, D.G. | Abdominal fat and insulin resistance in normal and overweight women: Direct measurements reveal a strong relationship in subjects at both low and high risk of NIDDM | Code 2 |
| Carey, D.G. | Abdominal fat and insulin resistance in normal and overweight women: Direct measurements reveal a strong relationship in subjects at both low and high risk of NIDDM | Code 1 |
| Carrick-Ranson, G. | The larger exercise stroke volume in endurance-trained men does not result from increased left ventricular early or late inflow or tissue velocities | Code 1 |
| Cartier, A. | Sex differences in inflammatory markers: what is the contribution of visceral adiposity? | Code 2 |
| Chang, A.Y. | Associations among androgens, estrogens, and natriuretic peptides in young women: observations from the Dallas Heart Study | Code 2 |
| Chen, C.H. | The study of anthropometric estimates in the visceral fat of healthy individuals | Code 1 |
| Chen, G.C. | Association between regional body fat and cardiovascular disease risk among postmenopausal women with normal body mass index | Code 3 |
| Cheng, S. | Adiposity, cardiometabolic risk, and vitamin D status: the Framingham Heart Study | Code 2 |
| Chitongo, P.B. | Visceral Adiposity Is an Independent Determinant of Hypercoagulability as Measured by Thrombin Generation in Morbid Obesity | Code 1 |
| Choi, J.W. | Bone mineral density correlates strongly with basal metabolic rate in postmenopausal women | Code 2 |
| Chowdhary, A. | Coronary microvascular function and visceral adiposity in patients with normal body weight and type 2 diabetes | Code 2 |
| Christoph, M.J. | Impact of adiposity on cellular adhesion: The Multi-Ethnic Study of atherosclerosis (MESA) | Code 2 |
| Chughtai, H.L. | Intraperitoneal fat is associated with thickening of the thoracic aorta in individuals at high risk for cardiovascular events | Code 2 |
| Coats, V. | Ectopic adiposity and cardiometabolic health in COPD | Code 2 |
| Coker, R.H. | Influence of exercise intensity on abdominal fat and adiponectin in elderly adults | Code 2 |
| Colica, C. | Efficacy and safety of very-low-calorie ketogenic diet: a double blind randomized crossover study | Code 3 |
| Conway, B. | Double-edged relationship between adiposity and coronary artery calcification in type 1 diabetes | Code 2 |
| Conway, J.M. | Intraabdominal adipose tissue and anthropometric surrogates in African American women with upper- and lower-body obesity | Code 1 |
| Couillard, C. | Apolipoprotein AI- and AI:AII-containing lipoproteins in white men and women of the HERITAGE Family Study: Associations with metabolic risk profile variables | Code 1 |
| Couillard, C. | Gender difference in postprandial lipemia: Importance of visceral adipose tissue accumulation | Code 1 |
| Crist, B.L. | Association of oxidative stress, iron, and centralized fat mass in healthy postmenopausal women | Code 1 |
| Cronin, O. | The association of visceral adiposity with cardiovascular events in patients with peripheral artery disease | Code 5 |
| Cursino, K. | Subclinical cardiovascular disease parameters after one year in new users of depot medroxyprogesterone acetate compared to copper-IUD | Code 3 |
| D. Appiah | The Association of Lactation Duration with Visceral and Pericardial Fat Volumes in Parous Women: The CARDIA Study | Code 1 |
| D. H. Lee | The utility of predicted values in place of directly measured body composition | Code 2 |
| D. H. Son | Association of the new visceral adiposity index with coronary artery calcification and arterial stiffness in Korean population | Code 2 |
| D. Hasic | Vertebral Bone Marrow Fat Is independently Associated to VAT but Not to SAT: KORA FF4-Whole-Body MR Imaging in a Population-Based Cohort | Code 2 |
| D. R. Laddu | DXA Versus Clinical Measures of Adiposity as Predictors of Cardiometabolic Diseases and All-Cause Mortality in Postmenopausal Women | Code 3 |
| Dabelea, D. | Effect of Type 1 Diabetes on the Gender Difference in Coronary Artery Calcification: A Role for Insulin Resistance?: The Coronary Artery Calcification in Type 1 Diabetes (CACTI) Study | Code 2 |
| Dao, H-H. | Abnormal body composition phenotypes in Vietnamese women with early rheumatoid arthritis | Code 2 |
| Davison, K. | Relationships between Obesity, Cardiorespiratory Fitness, and Cardiovascular Function | Code 2 |
| De Benedetto, F. | The role of nutritional status in the global assessment of severe COPD patients | Code 2 |
| De La Maza, M.P. | Skeletal muscle ceramide species in men with abdominal obesity | Code 2 |
| De Lorenzo, A. | Normal-weight obese syndrome: early inflammation? | Code 2 |
| De Lorenzo, A. | Adiposity rather than BMI determines metabolic risk | Code 2 |
| De Lorenzo, F. | Association of DEXA-assessed total body fat mass with serum lipids and haemostatic variables | Code 2 |
| De Lorenzo, F. | Chronic fatigue syndrome: physical and cardiovascular deconditioning | Code 2 |
| De Mutsert, R. | The Netherlands epidemiology of obesity (NEO) study: study design and data collection | Code 2 |
| Dehghan, A. | A prospective study of the relationships between change in body composition and cardiovascular risk factors across the menopause | Code 1 |
| Demerath, E.W. | Visceral adiposity and its anatomical distribution as predictors of the metabolic syndrome and cardiometabolic risk factor levels | Code 2 |
| Despres, J. P. | Adipose tissue distribution and plasma lipoprotein levels in obese women. Importance of intra-abdominal fat | Code 8 |
| Despres, J.P. | Loss of abdominal fat and metabolic response to exercise training in obese women | Code 2 |
| Di Carlo, C. | Serum leptin levels and body composition in postmenopausal women: effects of hormone therapy | Code 1 |
| Di Daniele, N. | Body composition changes and cardiometabolic benefits of a balanced Italian Mediterranean Diet in obese patients with metabolic syndrome | Code 1 |
| Diaz, A.A. | Abdominal Visceral Adipose Tissue is Associated with Myocardial Infarction in Patients with COPD | Code 2 |
| Ding, L. | The impact of fat distribution on subclinical coronary atherosclerosis in middle-aged Chinese adults | Code 1 |
| Direk, K. | The relationship between DXA-based and anthropometric measures of visceral fat and morbidity in women | Code 2 |
| Divers, J. | Regional adipose tissue associations with calcified atherosclerotic plaque: African American-diabetes heart study | Code 2 |
| Dobrosielski, D. | Associations between vasodilatory capacity, physical activity and sleep among younger and older adults | Code 2 |
| Dobson, R. | Metabolically healthy and unhealthy obesity: Differential effects on myocardial function according to metabolic syndrome, rather than obesity | Code 2 |
| Donini, L.M. | Disability, physical inactivity, and impaired health-related quality of life are not different in metabolically healthy vs. Unhealthy obese subjects | Code 2 |
| Dubé, M.C. | The contribution of visceral adiposity and mid-thigh fat-rich muscle to the metabolic profile in postmenopausal women | Code 2 |
| Duckers, J.M. | Cardiovascular and musculskeletal co-morbidities in patients with alpha 1 antitrypsin deficiency | Code 2 |
| Dvorak, R.V. | Phenotypic characteristics associated with insulin resistance in metabolically obese but normal-weight young women | Code 2 |
| E. Beller | Significant Impact of Coffee Consumption on MR-Based Measures of Cardiac Function in a Population-Based Cohort Study without Manifest Cardiovascular Disease | Code 3 |
| E. S. Lau | Cardiovascular Biomarkers of Obesity and Overlap With Cardiometabolic Dysfunction | Code 1 |
| E. S. Orwol | CT Muscle Density, D3Cr Muscle Mass, and Body Fat Associations With Physical Performance, Mobility Outcomes, and Mortality Risk in Older Men | Code 3 |
| Eastwood, S.V. | Thigh fat and muscle each contribute to excess cardiometabolic risk in South Asians, independent of visceral adipose tissue | Code 2 |
| Eguchi, M. | Visceral obesity in Japanese patients with metabolic syndrome: Reappraisal of diagnostic criteria by CT scan | Code 2 |
| Eklund, D. | Fitness, body composition and blood lipids following 3 concurrent strength and endurance training modes | Code 3 |
| El Khoudary, S.R. | Ectopic cardiovascular fat in middle-aged men: Effects of race/ethnicity, overall and central adiposity. The ERA JUMP study | Code 2 |
| Emmons, R.R. | The influence of visceral fat on the postprandial lipemic response in men with paraplegia | Code 1 |
| Erickson, M.L. | Effects of Lifestyle Intervention on Plasma Trimethylamine N-Oxide in Obese Adults | Code 1 |
| Evans, E.M. | Aerobic power and insulin action improve in response to endurance exercise training in healthy 77-87 yr olds | Code 3 |
| F. Magkos | A protein-supplemented very-low-calorie diet does not mitigate reductions in lean mass and resting metabolic rate in subjects with overweight or obesity: a randomized controlled trial | Code 4 |
| F. Morys | Association Between Midlife Obesity and Its Metabolic Consequences, Cerebrovascular Disease, and Cognitive Decline | Code 2 |
| F. V. C. Machado | Longitudinal changes in total and regional body composition in patients with chronic obstructive pulmonary disease | Code 1 |
| Fantin, F. | Abdominal obesity and subclinical vascular damage in the elderly | Code 1 |
| Farajian, P. | Obesity indices in relation to cardiovascular disease risk factors among young adult female students | Code 3 |
| Ferreira, F.C. | Circuit resistance training in women with normal weight obesity syndrome: body composition, cardiometabolic and echocardiographic parameters, and cardiovascular and skeletal muscle fitness | Code 1 |
| Fialho, A. | Higher visceral to subcutaneous fat ratio is associated with small intestinal bacterial overgrowth | Code 1 |
| Figueroa, A. | Relationship between Measures of Adiposity, Arterial Inflammation, and Subsequent Cardiovascular Events | Code 5 |
| Finch, P. | Intra-abdominal fat: Comparison of computed tomography fat segmentation and bioimpedance spectroscopy | Code 1 |
| Flack, K.D. | Resistance exercise training and in vitro skeletal muscle oxidative capacity in older adults | Code 4 |
| Forbang, N.I. | Associations of body composition measures and C2, a marker for small artery elasticity: The MESA | Code 2 |
| Fornari, R. | Insulin growth factor-1 correlates with higher bone mineral density and lower inflammation status in obese adult subjects | Code 2 |
| Foster, G.D. | A randomized trial of the effects of an almond-enriched, hypocaloric diet in the treatment of obesity | Code 3 |
| Fox, C.S. | Pericardial fat, intrathoracic fat, and measures of left ventricular structure and function: the Framingham Heart Study | Code 2 |
| Fox, C.S. | Abdominal visceral and subcutaneous adipose tissue compartments: association with metabolic risk factors in the Framingham Heart Study | Code 2 |
| Fox, C.S. | Genome-wide association of pericardial fat identifies a unique locus for ectopic fat | Code 1 |
| Frank, L.L. | Effects of exercise on metabolic risk variables in overweight postmenopausal women: a randomized clinical trial | Code 1 |
| Friesen, K.J. | Bone Mineral Density and Body Composition of Collegiate Modern Dancers | Code 1 |
| Fujimoto, W.Y. | Visceral adiposity and incident coronary heart disease in Japanese - American men: The 10-year follow-up results of the Seattle Japanese-American community diabetes study | Code 2 |
| Fujimoto, W.Y. | The visceral adiposity syndrome in Japanese-American men | Code 1 |
| Fujimoto, W.Y. | Visceral fat obesity and morbidity: NIDDM and atherogenic risk in Japanese American men and women | Code 1 |
| Fukazawa, K. | Continuous imaging of esophagogastric junction in patients with reflux esophagitis using 320-row area detector CT: A feasibility study | Code 1 |
| Fukuda, T. | Sarcopenic obesity assessed using dual energy X-ray absorptiometry (DXA) can predict cardiovascular disease in patients with type 2 diabetes: A retrospective observational study | Code 5 |
| G. C. Campos | Mortality, sarcopenic obesity, and sarcopenia: Frailty in Brazilian Older People Study - FIBRA - RJ | Code 3 |
| G. Haber | The perils of using predicted values in place of observed covariates: an example of predicted values of body composition and mortality risk | Code 3 |
| G. Huang | Effect of Protein Intake on Visceral Abdominal Fat and Metabolic Biomarkers in Older Men With Functional Limitations: Results From a Randomized Clinical Trial | Code 4 |
| Gaborit, B. | Assessment of epicardial fat volume and myocardial triglyceride content in severely obese subjects: Relationship to metabolic profile, cardiac function and visceral fat | Code 1 |
| Gadelha, A.B. | Severity of sarcopenia is associated with postural balance and risk of falls in community-dwelling older women | Code 2 |
| Gaillard, T. | Ethnic differences in serum lipids and lipoproteins in overweight/obese African-American and white American women with pre-diabetes: Significance of NMR-derived lipoprotein particle concentrations and sizes | Code 2 |
| Gao, Y. | Correlations between the abdominal fat-related parameters and severity of coronary artery disease assessed by computed tomography | Code 2 |
| Garg, S.K. | Ectopic Fat Depots and Coronary Artery Calcium in South Asians Compared With Other Racial/Ethnic Groups | Code 2 |
| Gepner, Y. | The beneficial effects of Mediterranean diet over low-fat diet may be mediated by decreasing hepatic fat content | Code 1 |
| Giger, J.N. | Behavioral risk reduction strategies for chronic indicators and high-risk factors for premenopausal AAW with a prevalence for CHD | Code 3 |
| Gill, C. | Association of Visceral Adipose Tissue and Subclinical Atherosclerosis in US-Born Mexican Americans but not First Generation Immigrants | Code 2 |
| Gill, C.M. | Sex differences in pericardial adipose tissue assessed by PET/CT and association with cardiometabolic risk | Code 2 |
| Goffredo, M. | Role of Gut Microbiota and Short Chain Fatty Acids in Modulating Energy Harvest and Fat Partitioning in Youth | Code 3 |
| Goh, V.H.H. | Association of general and abdominal obesity with age, endocrine and metabolic factors in Asian men | Code 2 |
| Gohbara, M. | Association between epicardial adipose tissue volume and myocardial salvage in patients with a first ST-segment elevation myocardial infarction: An epicardial adipose tissue paradox | Code 5 |
| Golledge, J. | Body mass index is inversely associated with mortality in patients with peripheral vascular disease | Code 5 |
| Gonçalves, F.B. | Body-mass index, abdominal adiposity, and cardiovascular risk...Lancet. 2011 Mar 26;377(9771):1085-95 | Code 1 |
| González-Reimers, E. | Prognostic value of nutritional status in alcoholics, assessed by double-energy X-ray absorptiometry | Code 3 |
| Graffy, P. M. | Automated assessment of longitudinal biomarker changes at abdominal CT: correlation with subsequent cardiovascular events in an asymptomatic adult screening cohort | Code 8 |
| Granér, M. | Biomarkers and prediction of myocardial triglyceride content in non-diabetic men | Code 2 |
| Granér, M. | Cardiac steatosis associates with visceral obesity in nondiabetic obese men | Code 2 |
| Granér, M. | Epicardial fat, cardiac dimensions, and low-grade inflammation in young adult monozygotic twins discordant for obesity | Code 2 |
| Greaves, K.A | Cholesteryl ester transfer protein and lecithin: Cholesterol acyltransferase activities in hispanic and anglo postmenopausal women: Associations with total and regional body fat | Code 2 |
| Greenfield, J.R. | Obesity is an important determinant of baseline serum C-reactive protein concentration in monozygotic twins, independent of genetic influences | Code 2 |
| Guenther, M. | Adiposity distribution influences circulating adiponectin levels | Code 2 |
| Gullaksen, S. | Volumes of coronary plaque disease in relation to body mass index, waist circumference, truncal fat mass and epicardial adipose tissue in patients with type 2 diabetes mellitus and controls | Code 2 |
| H. Lee | Clinical Significance of Body Fat Distribution in Coronary Artery Calcification Progression in Korean Population | Code 1 |
| H. Oh | Adiposity and mortality in Korean adults: a population-based prospective cohort study | Code 3 |
| H. Y. Park | Relationship Between Sarcopenia, Obesity, Osteoporosis, and Cardiometabolic Health Conditions and Physical Activity Levels in Korean Older Adults | Code 2 |
| Hall, M.E. | Adiposity is associated with gender-specific reductions in left ventricular myocardial perfusion during dobutamine stress | Code 2 |
| Hallsworth, K. | Modified high-intensity interval training reduces liver fat and improves cardiac function in non-alcoholic fatty liver disease: a randomized controlled trial | Code 1 |
| Han, S. J. | Change in visceral adiposity is an independent predictor of future arterial pulse pressure | Code 1 |
| Harada, Y. | Differences in associations between visceral fat accumulation and obstructive sleep apnea by sex | Code 2 |
| Hargens, T.A. | Attenuated heart rate recovery following exercise testing in overweight young men with untreated obstructive sleep apnea | Code 1 |
| Hasic, D. | Vertebral Bone Marrow Fat Is independently Associated to VAT but Not to SAT: KORA FF4—Whole-Body MR Imaging in a Population-Based Cohort | Code 2 |
| He, H. | Sex difference in cardiometabolic risk profile and adiponectin expression in subjects with visceral fat obesity | Code 2 |
| He, J. | Cardiometabolic risks during anabolic hormone supplementation in older men | Code 1 |
| Hegazi, R.A. | Relationship of adiposity to subclinical atherosclerosis in obese patients with type 2 diabetes | Code 1 |
| Henriksen, K. | Efficacy and safety of the PPAR partial agonist balaglitazone compared with pioglitazone and placebo: a phase III, randomized, parallel-group study in patients with type 2 diabetes on stable insulin therapy | Code 3 |
| Hetland, M.L. | Regional body composition determined by dual-energy X-ray absorptiometry. Relation to training, sex hormones, and serum lipids in male long-distance runners | Code 1 |
| Heywood, R. | Hearing Loss and Risk of Mild Cognitive Impairment and Dementia: Findings from the Singapore Longitudinal Ageing Study | Code 3 |
| Hill, A.M. | Combining fish-oil supplements with regular aerobic exercise improves body composition and cardiovascular disease risk factors | Code 3 |
| Hill, J.O. | Racial differences in amounts of visceral adipose tissue in young adults: the CARDIA (Coronary Artery Risk Development in Young Adults) study | Code 2 |
| Ho, J.S. | Comparative Relation of General, Central, and Visceral Adiposity Measures for Coronary Artery Calcium in Subjects Without Previous Coronary Events | Code 1 |
| Hoenig, M.R. | Liver fat percent is associated with metabolic risk factors and the metabolic syndrome in a high-risk vascular cohort | Code 2 |
| Hoenig, M.R. | Low density lipoprotein cholesterol is inversely correlated with abdominal visceral fat area: A magnetic resonance imaging study | Code 5 |
| Hong, N.S. | The association between obesity and mortality in the elderly differs by serum concentrations of persistent organic pollutants: a possible explanation for the obesity paradox | Code 3 |
| Horta, B.L. | Cohort profile update: the 1982 pelotas (brazil) birth cohort study | Code 2 |
| Hotta, K. | Replication study of 15 recently published loci for body fat distribution in the Japanese population | Code 1 |
| Hotta, K. | Genetic variations in the CYP17A1 and NT5C2 genes are associated with a reduction in visceral and subcutaneous fat areas in Japanese women | Code 3 |
| Hotta, K. | Computed tomography analysis of the association between the SH2B1 rs7498665 single-nucleotide polymorphism and visceral fat area | Code 1 |
| Houghton, D. | Effects of Exercise on Liver Fat and Metabolism in Alcohol Drinkers | Code 1 |
| Hsu, F-C. | Adiposity is inversely associated with hippocampal volume in African Americans and European Americans with diabetes | Code 5 |
| Huang, G. | Testosterone dose-response relationships with cardiovascular risk markers in androgen-deficient women: a randomized, placebo-controlled trial | Code 4 |
| Hull, H.R. | The effect of the holiday season on body weight and composition in college students | Code 1 |
| Hung, C.L. | An observational study of the association among interatrial adiposity by computed tomography measure, insulin resistance, and left atrial electromechanical disturbances in heart failure | Code 2 |
| Hunter, G.R. | Fat distribution and cardiovascular disease risk in African-American women | Code 1 |
| Hunter, G.R. | Fat distribution, physical activity, and cardiovascular risk factors | Code 1 |
| I. Y. Tian | Predicting 3D body shape and body composition from conventional 2D photography | Code 2 |
| Ichikawa, R. | Influencing factors on cardiac structure and function beyond glycemic control in patients with type 2 diabetes mellitus | Code 1 |
| Idoate, F. | Adipose tissue compartments, muscle mass, muscle fat infiltration, and coronary calcium in institutionalized frail nonagenarians | Code 5 |
| Idoate, F. | Weight-loss diet alone or combined with resistance training induces different regional visceral fat changes in obese women | Code 1 |
| Igase, M. | Anti-aging dock | Code 6 |
| Imamura, T. | [Relationship between abdominal fat distribution assessed by computed tomography and serum lipids in the elderly] | Code 6 |
| Irlbeck, T. | Association between single-slice measurements of visceral and abdominal subcutaneous adipose tissue with volumetric measurements: the Framingham Heart Study | Code 1 |
| Isaac, V. | Adverse Associations between Visceral Adiposity, Brain Structure, and Cognitive Performance in Healthy Elderly | Code 2 |
| Ishai, A. | Amygdalar Metabolic Activity Independently Associates With Progression of Visceral Adiposity | Code 1 |
| Iturriaga, T. | Effects of a short workplace exercise program on body composition in women: A randomized controlled trial | Code 4 |
| J. Gade | Comparison of a dual-frequency bio-impedance analyser with dual-energy X-ray absorptiometry for assessment of body composition in geriatric patients | Code 4 |
| J. L. Heileson | Can Total Body Composition Be Used As A Predictor Of Cardiorespiratory Fitness In The Absence Of Cardiometabolic Diseases?...2021 ACSM Annual Meeting & World Congresses [Virtual], June 1-5, 2021 | Code 1 |
| J. Liu | Cardiac remodeling and subclinical left ventricular dysfunction in adults with uncomplicated obesity: a cardiovascular magnetic resonance study | Code 2 |
| J. Zhu | Association of Periaortic Fat and Abdominal Visceral Fat with Coronary Artery Atherosclerosis in Chinese Middle Aged and Elderly Patients Undergoing Computed Tomography Coronary Angiography | Code 2 |
| Jacobsen, D.E. | Raloxifene and tibolone in elderly women: a randomized, double-blind, double-dummy, placebo-controlled trial | Code 3 |
| Jang, Y. | Differences in body fat distribution and antioxidant status in Korean men with cardiovascular disease with or without diabetes | Code 2 |
| Janiszewski, P.M. | Breast volume is an independent predictor of visceral and ectopic fat in premenopausal women | Code 2 |
| Jensen, E.X. | Impact of chronic cigarette smoking on body composition and fuel metabolism | Code 1 |
| Jensky, N.E. | The association between abdominal body composition and vascular calcification | Code 1 |
| Jentzsch, T. | Are the rib fracture score and different computed tomography measures of obesity predictors for mortality in patients with rib fractures? A retrospective cohort study | Code 5 |
| Ji, Y. | Genome-wide and abdominal MRI data provide evidence that a genetically determined favorable adiposity phenotype is characterized by lower ectopic liver fat and lower risk of type 2 diabetes, heart disease, and hypertension | Code 1 |
| Johnstone, A.M. | Effects of a high-protein, low-carbohydrate v. High-protein, moderate-carbohydrate weight-loss diet on antioxidant status, endothelial markers and plasma indices of the cardiometabolic profile | Code 3 |
| Jones, A. | Adiposity is associated with blunted cardiovascular, neuroendocrine and cognitive responses to acute mental stress | Code 1 |
| Jonker, J.T. | Pioglitazone compared with metformin increases pericardial fat volume in patients with type 2 diabetes mellitus | Code 2 |
| K. A. Schmidt | Clinical Intervention to Reduce Dietary Sugar does not Impact Liver Fat in Latino Youth, Regardless of PNPLA3 Genotype: a Randomized Controlled Trial | Code 4 |
| K. Machado | Risk Factors for Low Muscle Mass in a Population-based Prospective Cohort of Brazilian Community-dwelling Older Women: The Sao Paulo Ageing & Health (SPAH) Study | Code 1 |
| K. Meredith-Jones | Age- and sex-specific visceral fat reference cutoffs and their association with cardio-metabolic risk | Code 2 |
| K. Otagiri | Predictive Value of Abdominal Fat Distribution on Coronary Artery Disease Severity Stratified by Computed Tomography-Derived SYNTAX Score | Code 2 |
| K. Takahari | Impact of the distribution of epicardial and visceral adipose tissue on left ventricular diastolic function | Code 2 |
| Kabakambira, J.D. | Do current guidelines for waist circumference apply to black Africans? Prediction of insulin resistance by waist circumference among Africans living in America | Code 2 |
| Kahl, K.G. | Reduced muscle mass in middle-aged depressed patients is associated with male gender and chronicity | Code 2 |
| Kahl, K.G. | Effects of additional exercise training on epicardial, intra-abdominal and subcutaneous adipose tissue in major depressive disorder: A randomized pilot study | Code 2 |
| Kahl, K.G. | Pericardial, intra-abdominal, and subcutaneous adipose tissue in patients with major depressive disorder | Code 1 |
| Kahl, K.G. | Visceral fat deposition and insulin sensitivity in depressed women with and without comorbid borderline personality disorder | Code 1 |
| Kamath, S.K. | Cardiovascular disease risk factors in 2 distinct ethnic groups: Indian and Pakistani compared with American premenopausal women | Code 2 |
| Kammerlander, A. A. | Sex Differences in the Associations of Visceral Adipose Tissue and Cardiometabolic and Cardiovascular Disease Risk: The Framingham Heart Study | Code 1 |
| Kanaya, A.M. | Mediators of Atherosclerosis in South Asians Living in America (MASALA) study: Objectives, methods, and cohort description | Code 2 |
| Kaplan, D. | Research digest | Code 7 |
| Kardassis, D. | Impact of body composition, fat distribution and sustained weight loss on cardiac function in obesity | Code 1 |
| Karelis, A.D. | Relationship between the bertin index to estimate visceral adipose tissue from dual-energy x-ray absorptiometry and cardiometabolic risk factors before and after weight loss | Code 2 |
| Kataoka, T. | Relationship between epicardial adipose tissue volume and coronary artery spasm | Code 1 |
| Katsoulis, K. | Diet-induced changes in intra-abdominal adipose tissue and CVD risk in American women | Code 2 |
| Katsuki, A. | Increased oxidative stress is associated with decreased circulating levels of adiponectin in Japanese metabolically obese, normal-weight men with normal glucose tolerance | Code 1 |
| Katz, P. | Obesity and its measurement in a community-based sample of women with systemic lupus erythematosus | Code 2 |
| Katzmarzyk, P.T. | Clinical utility and reproducibility of visceral adipose tissue measurements derived from dual-energy X-ray absorptiometry in White and African American adults | Code 4 |
| Katzmarzyk, P.T. | Clinical utility of visceral adipose tissue for the identification of cardiometabolic risk in white and African American adults | Code 2 |
| Katzmarzyk, P.T. | Racial differences in abdominal depot-specific adiposity in white and African American adults | Code 2 |
| Kaul, S. | Dual-energy X-ray absorptiometry for quantification of visceral fat | Code 2 |
| Kaur, S. | Higher visceral fat is associated with lower cerebral N-acetyl-aspartate ratios in middle-aged adults | Code 1 |
| Kawamoto, R. | Metabolic syndrome as a predictor of ischemic stroke in elderly persons | Code 2 |
| Kazlauskaite, R. | Abdominal adiposity change in white and black midlife women: The study of women's health across the nation | Code 1 |
| Keating, S.E. | Continuous Exercise but Not High Intensity Interval Training Improves Fat Distribution in Overweight Adults | Code 2 |
| Kemmler, W. | Exercise, Body Composition, and Functional Ability. A Randomized Controlled Trial | Code 2 |
| Kensara, O.A. | Fetal programming of body composition: relation between birth weight and body composition measured with dual-energy X-ray absorptiometry and anthropometric methods in older Englishmen | Code 2 |
| Keogh, J.B. | The effect of meal replacements high in glycomacropeptide on weight loss and markers of cardiovascular disease risk [corrected] [published erratum appears in AM J CLIN NUTR 2008 Sep;88(3):810] | Code 3 |
| Khashper, A. | Visceral abdominal adipose tissue and coronary atherosclerosis in asymptomatic diabetics | Code 1 |
| Kikuchi, Y. | Effects of Whole Grain Wheat Bread on Visceral Fat Obesity in Japanese Subjects: A Randomized Double-Blind Study | Code 2 |
| Kim, D. | Nonalcoholic fatty liver disease is associated with coronary artery calcification | Code 2 |
| Kim, D.J. | Visceral adiposity and subclinical coronary artery disease in elderly adults: Rancho Bernardo study | Code 2 |
| Kim, H. J. | A Genome-wide association study on abdominal adiposity-related traits in adult Korean men | Code 2 |
| Kim, H.J. | A pilot study exploring the efficacy and safety of herbal medicine on Korean obese women with metabolic syndrome risk factors: Double blinded, randomized, multicenter, placebo controlled study protocol clinical trial | Code 4 |
| Kim, K.W. | Visceral obesity is associated with white matter hyperintensity and lacunar infarct | Code 2 |
| Kim, M.K. | The association between ectopic fat in the pancreas and subclinical atherosclerosis in type 2 diabetes | Code 2 |
| Kim, N.H. | Association of obstructive sleep apnea and glucose metabolism in subjects with or without obesity | Code 2 |
| Kim, S. | Body Composition and Physical Function in Older Adults with Various Comorbidities | Code 2 |
| Kim, S. | Normal-weight obesity is associated with increased risk of subclinical atherosclerosis | Code 2 |
| Kim, S. | Combined impact of cardiorespiratory fitness and visceral adiposity on metabolic syndrome in overweight and obese adults in Korea | Code 2 |
| Kim, S.H. | The associations of epicardial adipose tissue with coronary artery disease and coronary atherosclerosis | Code 2 |
| Kishida, K. | Relationships between circulating adiponectin levels and fat distribution in obese subjects | Code 1 |
| Knoepfli-Lenzin, C. | Effects of a 12-week intervention period with football and running for habitually active men with mild hypertension | Code 4 |
| Ko, G. | Abdominal Adiposity, Not Cardiorespiratory Fitness, Mediates the Exercise-Induced Change in Insulin Sensitivity in Older Adults | Code 4 |
| Kobayashi, H. | Visceral fat accumulation contributes to insulin resistance, small-sized low-density lipoprotein, and progression of coronary artery disease in middle-aged non-obese Japanese men | Code 2 |
| Kobayashi, J. | Effect of apolipoprotein E3/4 phenotype on postprandial triglycerides and retinyl palmitate metabolism in plasma from hyperlipidemic subjects in Japan | Code 1 |
| Kohno, M. | Decreases in serum triacylglycerol and visceral fat mediated by dietary soybean beta-conglycinin | Code 2 |
| Krakauer, J.C. | Body composition profiles derived from dual-energy X-ray absorptiometry, total body scan, and mortality | Code 3 |
| Kramer, C.K. | A prospective study of abdominal obesity and coronary artery calcium progression in older adults | Code 1 |
| Kueht, M.L. | Severely obese have greater LPS-stimulated TNF-alpha production than normal weight African-American women | Code 1 |
| Kuipers, A.L. | Association of ectopic fat with abdominal aorto-illiac and coronary artery calcification in african ancestry men | Code 3 |
| Kuk, J.L. | Body mass index and hip and thigh circumferences are negatively associated with visceral adipose tissue after control for waist circumference | Code 1 |
| Kuller, L.H. | The clinical trial of Women On the Move through Activity and Nutrition (WOMAN) study | Code 3 |
| Kunita, E. | Association between plasma high-molecular-weight adiponectin and coronary plaque characteristics assessed by computed tomography angiography in conditions of visceral adipose accumulation | Code 2 |
| Kwon, K. | Reciprocal association between visceral obesity and adiponectin: In healthy premenopausal women | Code 1 |
| L. J. Brandon | Do the Same Central Anthropometric Variables that Best Predict Blood Pressure in European Americans also Best Predict Blood Pressure in African Americans? | Code 2 |
| L. Lind | Cardiovascular-related proteins and the abdominal visceral to subcutaneous adipose tissue ratio | Code 1 |
| L. Shuo | Associations Between Vitamin D Level, Cardiovascular Risk Factors And Body Composition In Older Women...2021 ACSM Annual Meeting & World Congresses [Virtual], June 1-5, 2021 | Code 2 |
| Labbé, S.M. | Increased myocardial uptake of dietary fatty acids linked to cardiac dysfunction in glucose-intolerant humans | Code 1 |
| Ladeiras-Lopes, R. | The Ratio Between Visceral and Subcutaneous Abdominal Fat Assessed by Computed Tomography Is an Independent Predictor of Mortality and Cardiac Events | Code 8 |
| Lalande, S. | Left Ventricular Mass in Elite Olympic Weight Lifters | Code 1 |
| Lampe, L. | Visceral obesity relates to deep white matter hyperintensities via inflammation | Code 2 |
| Larsen, B. | Muscle area and density and risk of all-cause mortality: The Multi-Ethnic Study of Atherosclerosis | Code 3 |
| Larsen, B.A. | Adipokines and severity and progression of coronary artery calcium: Findings from the Rancho Bernardo Study | Code 1 |
| Larsen, B.A. | Pericardial fat is associated with all-cause mortality but not incident CVD: The Rancho Bernardo Study | Code 8 |
| Larsen, B.A. | Associations of physical activity and sedentary behavior with regional fat deposition | Code 2 |
| Lavie, C.J. | Body composition and heart failure prevalence and prognosis: Getting to the fat of the matter in the "obesity paradox" | Code 2 |
| Lazar, A.S. | Sleep deficits but no metabolic deficits in premanifest Huntington's disease | Code 2 |
| Lê, K.A. | Ethnic differences in pancreatic fat accumulation and its relationship with other fat depots and inflammatory markers | Code 2 |
| Lear, S.A. | Elevation in cardiovascular disease risk in South asians is mediated by differences in visceral adipose tissue | Code 2 |
| Lear, S.A. | Oxidative stress is associated with visceral adipose tissue and subclinical atherosclerosis in a healthy multi-ethnic population | Code 2 |
| Lee, C.Y. | Correlation between executive network integrity and sarcopenia in patients with parkinson’s disease | Code 2 |
| Lee, E.S. | Depressive mood and abdominal fat distribution in overweight premenopausal women | Code 2 |
| Lee, H. | Clinical Significance of Body Fat Distribution in Coronary Artery Calcification Progression in Korean Population | Code 1 |
| Lee, I.T. | Brain-derived neurotrophic factor not associated with metabolic syndrome but inversely correlated with vascular cell adhesion molecule-1 in men without diabetes | Code 2 |
| Lee, J.J. | Longitudinal Associations of Pericardial and Intrathoracic Fat With Progression of Coronary Artery Calcium (from the Framingham Heart Study) | Code 1 |
| Lee, J.J. | Visceral and Intrahepatic Fat Are Associated with Cardiometabolic Risk Factors Above Other Ectopic Fat Depots: The Framingham Heart Study | Code 1 |
| Lee, J.J. | Relation of Iliac Artery Calcium With Adiposity Measures and Peripheral Artery Disease | Code 1 |
| Lee, J.J. | Association of Changes in Abdominal Fat Quantity and Quality With Incident Cardiovascular Disease Risk Factors | Code 1 |
| Lee, J.S.W. | Survival benefit of abdominal adiposity: A 6-year follow-up study with Dual X-ray absorptiometry in 3,978 older adults | Code 3 |
| Lee, S-Y. | The impact of obesity on subclinical coronary atherosclerosis according to the risk of cardiovascular disease | Code 2 |
| Lee, S. | Cardiorespiratory fitness attenuates metabolic risk independent of abdominal subcutaneous and visceral fat in men | Code 2 |
| Lee, Y.A. | Association between metabolic syndrome, smoking status and coronary artery calcification | Code 3 |
| Lemieux, I. | Is the gender difference in LDL size explained by the metabolic complications of visceral obesity? | Code 1 |
| Lesser, I.A. | Association between exercise-induced change in body composition and change in cardiometabolic risk factors in postmenopausal South Asian women | Code 4 |
| Lesser, I.A. | Effectiveness of Exercise on Visceral Adipose Tissue in Older South Asian Women | Code 2 |
| Lewis, T.T. | Hostility is associated with visceral, but not subcutaneous, fat in middle-aged african American and white women | Code 2 |
| Li, H-X. | Neck circumference as a measure of neck fat and abdominal visceral fat in Chinese adults | Code 1 |
| Liang, K.W. | MRI measured epicardial adipose tissue thickness at the right AV groove differentiates inflammatory status in obese men with metabolic syndrome | Code 1 |
| Liao, D. | Abnormal glucose tolerance and increased risk for cardiovascular disease in Japanese-Americans with normal fasting glucose | Code 4 |
| Lim, S. | Subclinical atherosclerosis in a community-based elderly cohort: The Korean Longitudinal Study on Health and Aging | Code 1 |
| Lima, W.A. | Body fat topography as a predictor of an increase in blood lipids | Code 1 |
| Linge, J. | Sub-phenotyping Metabolic Disorders Using Body Composition: An Individualized, Nonparametric Approach Utilizing Large Data Sets | Code 2 |
| Linge, J. | Body Composition Profiling in the UK Biobank Imaging Study | Code 2 |
| Liu, J. | Fatty liver, abdominal adipose tissue and atherosclerotic calcification in African Americans: the Jackson Heart Study | Code 2 |
| Liu, J. | Impact of abdominal visceral and subcutaneous adipose tissue on cardiometabolic risk factors: the Jackson Heart Study | Code 2 |
| Liu, J. | Pericardial adipose tissue, atherosclerosis, and cardiovascular disease risk factors: the Jackson heart study | Code 2 |
| Liu, P.Y. | Evidence for the association between abdominal fat and cardiovascular risk factors in overweight and obese African American women | Code 2 |
| Liu, X. | Blood urea nitrogen is elevated in patients with non-alcoholic fatty liver disease | Code 3 |
| Liu, Z.M. | Dietary sugar intake was associated with increased body fatness but decreased cardiovascular mortality in Chinese elderly: An 11-year prospective study of Mr and Ms OS of Hong Kong | Code 3 |
| Lo, J. | Effects of obesity, body composition, and adiponectin on carotid intima-media thickness in healthy women | Code 2 |
| Loenneke, J.P. | Quality protein intake is inversely related with abdominal fat | Code 2 |
| Loh, R.K.C. | Does chronic treatment with a thiazolidinedione increase brown fat thermogenesis in humans? | Code 2 |
| Looker, A.C. | Dysmobility syndrome and mortality risk in US men and women age 50 years and older | Code 3 |
| Lopez-Padros,C. | Effectiveness of an Intensive Weight-Loss Program for Severe Obstructive Sleep Apnea Syndrome (OSA) in Patients Undergoing CPAP Treatment: a Randomized Controlled Trial | Code 2 |
| Lopez-Padros,C. | Effectiveness of an Intensive Weight-Loss Program for Severe Obstructive Sleep Apnea Syndrome (OSA) in Patients Undergoing CPAP Treatment: a Randomized Controlled Trial | Code 3 |
| López-Reyes, A. | The HIF1A rs2057482 polymorphism is associated with risk of developing premature coronary artery disease and with some metabolic and cardiovascular risk factors. The Genetics of Atherosclerotic Disease (GEA) Mexican Study | Code 1 |
| Lorbeer, R. | Correlation of MRI-derived adipose tissue measurements and anthropometric markers with prevalent hypertension in the community | Code 2 |
| Lotscher, F. | Biologically relevant sex differences for fitness-related parameters in active octogenarians | Code 3 |
| Lotta, L.A. | Association of Genetic Variants Related to Gluteofemoral vs Abdominal Fat Distribution with Type 2 Diabetes, Coronary Disease, and Cardiovascular Risk Factors | Code 2 |
| Ludescher, B. | Increase of visceral fat and adrenal gland volume in women with depression: preliminary results of a morphometric MRI study | Code 1 |
| Lui, M.M. | C-reactive protein is associated with obstructive sleep apnea independent of visceral obesity | Code 2 |
| Lustig, R.H. | A multicenter, randomized, double-blind, placebo-controlled, dose-finding trial of a long-acting formulation of octreotide in promoting weight loss in obese adults with insulin hypersecretion | Code 1 |
| Lynch, N.A. | Comparison of VO2max and disease risk factors between perimenopausal and postmenopausal women | Code 2 |
| M. A. Refaee | The Linkage Between Bone Densitometry and Cardiovascular Disease...19th Annual International Conference on Informatics, Management, and Technology in Healthcare (ICIMTH), October 16-17, 2021 (Virtual) | Code 2 |
| M. Chlabicz | A similar lifetime cv risk and a similar cardiometabolic profile in the moderate and high cardiovascular risk populations: A population-based study | Code 2 |
| M. D. R. Klarqvist | Estimating body fat distribution - a driver of cardiometabolic health - from silhouette images | Code 3 |
| M. J. Galvan | Four weeks of electrical stimulation improves glucose tolerance in a sedentary overweight or obese Hispanic population | Code 4 |
| M. Ponce-de-Leon | Novel associations between inflammation-related proteins and adiposity: A targeted proteomics approach across four population-based studies | Code 2 |
| M. Wiecek | Whole-body cryotherapy is an effective method of reducing abdominal obesity in menopausal women with metabolic syndrome | Code 1 |
| Machado, K. | Risk Factors for Low Muscle Mass in a Population-based Prospective Cohort of Brazilian Community-dwelling Older Women: The Sao Paulo Ageing & Health (SPAH) Study | Code 1 |
| Machado, K. | Risk Factors for Low Muscle Mass in a Population-based Prospective Cohort of Brazilian Community-dwelling Older Women: The Sao Paulo Ageing & Health (SPAH) Study | Code 1 |
| Macpherson, R.E.K. | Run Sprint Interval Training Improves Aerobic Performance but Not Maximal Cardiac Output | Code 4 |
| Maersk, M. | Sucrose-sweetened beverages increase fat storage in the liver, muscle, and visceral fat depot: a 6-mo randomized intervention study | Code 4 |
| Mahabadi, A.A. | Association of pericardial fat, intrathoracic fat, and visceral abdominal fat with cardiovascular disease burden: The Framingham Heart Study | Code 2 |
| Major, G.C. | Energy expenditure from physical activity and the metabolic risk profile at menopause | Code 2 |
| Malavazos, A.E. | Monocyte chemoattractant protein 1: a possible link between visceral adipose tissue-associated inflammation and subclinical echocardiographic abnormalities in uncomplicated obesity | Code 1 |
| Manios, G.E. | Are obesity indices derived by dual-energy X-ray absorptiometry capable of identifying postmenopausal females with high risk for coronary heart disease? | Code 5 |
| Manios, G.E. | Abdominal fat volume estimation by stereology on CT: a comparison with manual planimetry | Code 1 |
| Marinou, K. | Structural and functional properties of deep abdominal subcutaneous adipose tissue explain its association with insulin resistance and cardiovascular risk in men | Code 2 |
| Markovic, T.P. | Beneficial effect on average lipid levels from energy restriction and fat loss in obese individuals with or without type 2 diabetes | Code 1 |
| Massalou, D. | Three-dimensional variability of the mesentery and the superior mesenteric artery: Application to virtual trauma modeling | Code 1 |
| Mayr, H.L. | Ad libitum Mediterranean diet reduces subcutaneous but not visceral fat in patients with coronary heart disease: A randomised controlled pilot study | Code 5 |
| McGrath, S. | Physical activity and central adiposity in a cohort of African-American adults | Code 2 |
| Menni, C. | Metabolomic profiling to dissect the role of visceral fat in cardiometabolic health | Code 2 |
| Micklesfield, L.K. | Dual-energy X-ray performs as well as clinical computed tomography for the measurement of visceral fat | Code 1 |
| Micklesfield, L.K. | Dual-energy X-ray absorptiometry and anthropometric estimates of visceral fat in Black and White South African Women | Code 1 |
| Miljkovic, I. | Greater Skeletal Muscle Fat Infiltration Is Associated With Higher All-Cause and Cardiovascular Mortality in Older Men | Code 5 |
| Miller, B.S. | A quantitative tool to assess degree of sarcopenia objectively in patients with hypercortisolism | Code 5 |
| Miranda, E.R. | Endogenous secretory RAGE increases with improvements in body composition and is associated with markers of adipocyte health | Code 1 |
| Mitsutake, R. | Are metabolic factors associated with coronary artery stenosis on MDCT? | Code 2 |
| Moeller, L.E. | Isoflavone-rich soy protein prevents loss of hip lean mass but does not prevent the shift in regional fat distribution in perimenopausal women | Code 3 |
| Momesso, D.P. | Increased epicardial adipose tissue in type 1 diabetes is associated with central obesity and metabolic syndrome | Code 2 |
| Mongraw-Chaffin, M.L. | Association between sex hormones and adiposity: qualitative differences in women and men in the multi-ethnic study of atherosclerosis | Code 1 |
| Moon, S.S. | Association between blood mercury level and visceral adiposity in adults | Code 2 |
| Muka, T. | Dietary fat composition, total body fat and regional body fat distribution in two Caucasian populations of middle-aged and older adult women | Code 3 |
| Munger, E. | Application of machine learning to determine top predictors of noncalcified coronary burden in psoriasis: An observational cohort study | Code 1 |
| Munger, E. | Application of machine learning to determine top predictors of noncalcified coronary burden in psoriasis: An observational cohort study | Code 1 |
| Murai, T. | Association of epicardial adipose tissue with serum level of cystatin C in type 2 diabetes | Code 2 |
| Murphy, R.A. | Adipose tissue, muscle, and function: Potential mediators of associations between body weight and mortality in older adults with type 2 diabetes | Code 1 |
| Mzayek, F. | Impact of Abdominal Obesity on Proximal and Distal Aorta Wall Thickness in African Americans: The Jackson Heart Study | Code 1 |
| N. E. Antonio-Villa | Increased visceral fat accumulation modifies the effect of insulin resistance on arterial stiffness and hypertension risk | Code 2 |
| N. Ozato | Association between Visceral Fat and Brain Structural Changes or Cognitive Function | Code 2 |
| N. Sawada | Independent effect of visceral fat on left atrial phasic function in the general population | Code 2 |
| N. Sawada | Influence of visceral adiposity accumulation on adverse left and right ventricular mechanics in the community | Code 2 |
| N. Schweighofer | DXA-Derived Indices in the Characterisation of Sarcopenia | Code 1 |
| N. Y. Krakauer | Association of X-ray Absorptiometry Body Composition Measurements with Basic Anthropometrics and Mortality Hazard | Code 3 |
| NA | Visualizing the Future | Code 2 |
| NA | The effects of ergometer intervention combining physical and cognitive training in older adults with mild cognitive impairment (MCI): a randomized control trial | Code 3 |
| Nakanishi-Minami, T. | Carotid intima-media thickness, but not visceral fat area or adiponectin, correlates with intracoronary stenosis detected by multislice computed tomography in people with type 2 diabetes and hypertension | Code 1 |
| Nakatsuji, H. | Hyperinsulinemia correlates with low levels of plasma B-type natriuretic peptide in Japanese men irrespective of fat distribution | Code 2 |
| Nalini, M. | Comparing anthropometric indicators of visceral and general adiposity as determinants of overall and cardiovascular mortality | Code 8 |
| Needham, B.L. | Endogenous sex steroid hormones and glucose in a South-Asian population without diabetes: the Metabolic Syndrome and Atherosclerosis in South-Asians Living in America pilot study | Code 2 |
| Neeland, I.J. | Dysfunctional adiposity and the risk of prediabetes and type 2 diabetes in obese adults | Code 1 |
| Nicklas, B.J. | Association of Visceral Adipose Tissue with Incident Myocardial Infarction in Older Men and Women | Code 1 |
| Nicklas, B.J. | Abdominal obesity is an independent risk factor for chronic heart failure in older people | Code 1 |
| Nicklas, B.J. | Lifestyle intervention of hypocaloric dieting and walking reduces abdominal obesity and improves coronary heart disease risk factors in obese, postmenopausal, African-American and Caucasian women | Code 1 |
| Nicklas, B.J. | Visceral adipose tissue cutoffs associated with metabolic risk factors for coronary heart disease in women | Code 1 |
| Niemiro, G.M. | Oral Glucose Tolerance is Associated with Neuroelectric Indices of Attention Among Adults with Overweight and Obesity | Code 2 |
| Niwa, Y. | Association between stroke and metabolic syndrome in a Japanese population: Jichi Medical School (JMS) Cohort Study | Code 3 |
| Nyman, K. | Metabolic syndrome associates with left atrial dysfunction | Code 2 |
| O. O | Defining cutoffs to diagnose obesity using the relative fat mass (RFM): Association with mortality in NHANES 1999–2014 | Code 1 |
| Odegaard, A. O. | Weight and mortality: Why body composition matters | Code 2 |
| Oh, H. | Adiposity and mortality in Korean adults: a population-based prospective cohort study | Code 3 |
| Oh, S.K. | Derivation and validation of a new visceral adiposity index for predicting visceral obesity and cardiometabolic risk in a Korean population | Code 2 |
| Oikawa, M. | Predominance of Abdominal Visceral Adipose Tissue Reflects the Presence of Aortic Valve Calcification | Code 5 |
| Oikawa, M. | Epicardial adipose tissue reflects the presence of coronary artery disease: Comparison with abdominal visceral adipose tissue | Code 1 |
| Okada, K. | Epicardial fat volume correlates with severity of coronary artery disease in nonobese patients | Code 1 |
| Okazaki, M. | Identification of unique lipoprotein subclasses for visceral obesity by component analysis of cholesterol profile in high-performance liquid chromatography | Code 1 |
| Okura, T. | Regional Body Composition Changes Exhibit Opposing Effects on Coronary Heart Disease Risk Factors | Code 1 |
| Okura, T. | Effects of exercise intensity on physical fitness and risk factors for coronary heart disease | Code 1 |
| Okura, T. | Relationships of resting energy expenditure with body fat distribution and abdominal fatness in Japanese population | Code 1 |
| Okura, T. | Effects of obesity phenotype on coronary heart disease risk factors in response to weight loss | Code 1 |
| Oliveira, A.L. | Visceral and subcutaneous adipose tissue FDG uptake by PET/CT in metabolically healthy obese subjects | Code 1 |
| Onat, A. | Visceral adipose tissue and body fat mass: Predictive values for and role of gender in cardiometabolic risk among Turks | Code 2 |
| Onat, A. | Measures of abdominal obesity assessed for visceral adiposity and relation to coronary risk | Code 2 |
| Osawa, K. | Nonalcoholic hepatic steatosis is a strong predictor of high-risk coronary-artery plaques as determined by multidetector CT | Code 2 |
| Osawa, K. | Differential association of visceral adipose tissue with coronary plaque characteristics in patients with and without diabetes mellitus | Code 1 |
| P. K. Bhanu | CAFT: a deep learning-based comprehensive abdominal fat analysis tool for large cohort studies | Code 1 |
| P. M. | Body Weight, BMI, Percent Fat and Associations with Mortality and Incident Mobility Limitation in Older Men | Code 3 |
| P. Natarajan | Chromosome Xq23 is associated with lower atherogenic lipid concentrations and favorable cardiometabolic indices | Code 1 |
| P. Srikanthan | Sex Differences in the Association of Body Composition and Cardiovascular Mortality | Code 3 |
| Paradis, M.E. | Visceral adipose tissue accumulation, secretory phospholipase A 2-IIA and atherogenecity of LDL | Code 1 |
| Parikh, N.I. | Visceral and subcutaneous adiposity and brachial artery vasodilator function | Code 1 |
| Park, H. | Prevalence and risk factors of cerebral white matter changes and silent infarcts on brain computed tomography scans among community-dwelling healthy adults: The PRESENT project | Code 2 |
| Park, H.S. | Postmenopausal women lose less visceral adipose tissue during a weight reduction program | Code 1 |
| Park, J.K. | Body Fat Distribution After Menopause and Cardiovascular Disease Risk Factors: Korean National Health and Nutrition Examination Survey 2010 | Code 1 |
| Park, S.J. | The relationship between coronary atherosclerosis and body fat distribution measured using dual energy X-ray absorptiometry | Code 2 |
| Park, Y.W. | Larger amounts of visceral adipose tissue in Asian Americans | Code 1 |
| Parker, B. | Effect of a high-protein, high-monounsaturated fat weight loss diet on glycemic control and lipid levels in type 2 diabetes | Code 3 |
| Pascot, A. | Deterioration of the metabolic risk profile in women. Respective contributions of impaired glucose tolerance and visceral fat accumulation | Code 1 |
| Pataky, Z. | Impact of Hypocaloric Hyperproteic Diet on Gut Microbiota in Overweight or Obese Patients with Nonalcoholic Fatty Liver Disease: A Pilot Study | Code 1 |
| Patel, V.G. | Left Ventricular Function Across the Spectrum of Body Mass Index in African Americans: The Jackson Heart Study | Code 2 |
| Pavithran, N. | The Effect of a Low GI Diet on Truncal Fat Mass and Glycated Hemoglobin in South Indians with Type 2 Diabetes—A Single Centre Randomized Prospective Study | Code 1 |
| Peiris, A.N. | Relationship of regional fat distribution and obesity to electrocardiographic parameters in healthy premenopausal women | Code 1 |
| Peppa, M. | Body composition determinants of metabolic phenotypes of obesity in nonobese and obese postmenopausal women | Code 2 |
| Peppa, M. | Association of lean body mass with cardiometabolic risk factors in healthy postmenopausal women | Code 2 |
| Perrone, M.A. | Effects of postprandial hydroxytyrosol and derivates on oxidation of LDL, cardiometabolic state and gene expression: A nutrigenomic approach for cardiovascular prevention | Code 3 |
| Perry, C.D. | Centrally located body fat is related to inflammatory markers in healthy postmenopausal women | Code 2 |
| Peterson, C.M. | Effect of 12 wk of resistant starch supplementation on cardiometabolic risk factors in adults with prediabetes: a randomized controlled trial | Code 2 |
| Picardi, A. | Association between non-alcoholic fatty liver disease and cardiovascular disease: A first message should pass | Code 2 |
| Pickhardt, P.J. | Automated CT biomarkers for opportunistic prediction of future cardiovascular events and mortality in an asymptomatic screening population: a retrospective cohort study | Code 3 |
| Piernas, C. | New computed tomography-derived indices to predict cardiovascular and insulin-resistance risks in overweight/obese patients | Code 1 |
| Pinnick, K.E. | Distinct developmental profile of lower-body adipose tissue defines resistance against obesity-associated metabolic complications | Code 2 |
| Pintér, Z. | Anthropometric dimensions provide reliable estimates of abdominal adiposity: A validation study | Code 1 |
| Playford, M.P. | Serum active 1,25(OH)<sub>2</sub>D, but not inactive 25(OH)D vitamin D levels are associated with cardiometabolic and cardiovascular disease risk in psoriasis | Code 3 |
| Porter, S.A. | Abdominal subcutaneous adipose tissue: a protective fat depot? | Code 1 |
| Preis, S.R. | Abdominal subcutaneous and visceral adipose tissue and insulin resistance in the Framingham heart study | Code 1 |
| Prior, S.J. | Genetic and environmental influences on skeletal muscle phenotypes as a function of age and sex in large, multigenerational families of African heritage | Code 3 |
| Pritchard, J.E. | Benefits of a year-long workplace weight loss program on cardiovascular risk factors | Code 1 |
| Prokop, N.W. | Do Canadian collegiate hockey players accurately perceive body composition changes after unmonitored training and diet? | Code 3 |
| Pugh, C.J.A. | Exercise training improves cutaneous microvascular function in nonalcoholic fatty liver disease | Code 1 |
| Punyadeera, C. | Ethnic differences in lipid metabolism in two groups of obese South African women | Code 1 |
| Q. Qin | Bioelectrical impedance analysis versus quantitative computer tomography and anthropometry for the assessment of body composition parameters in China | Code 2 |
| R. T. Hurt | The Comparison of Segmental Multifrequency Bioelectrical Impedance Analysis and Dual-Energy X-ray Absorptiometry for Estimating Fat Free Mass and Percentage Body Fat in an Ambulatory Population | Code 1 |
| R. von Krüchten | Association between Adipose Tissue Depots and Dyslipidemia: The KORA-MRI Population-Based Study | Code 2 |
| R. Wagner | Pathophysiology-based subphenotyping of individuals at elevated risk for type 2 diabetes | Code 2 |
| Räikkönen, K. | Anger, hostility, and visceral adipose tissue in healthy postmenopausal women | Code 1 |
| Raja, G.K. | Commonality versus specificity among adiposity traits in normal-weight and moderately overweight adults | Code 1 |
| Rajaram, S. | The Walnuts and Healthy Aging Study (WAHA): protocol for a nutritional intervention trial with walnuts on brain aging | Code 1 |
| Raji, A. | Body fat distribution and insulin resistance in healthy Asian Indians and Caucasians | Code 2 |
| Ramprasath, V.R. | Effect of consuming novel foods consisting high oleic canola oil, barley β-glucan, and DHA on cardiovascular disease risk in humans: the CONFIDENCE (Canola Oil and Fibre with DHA Enhanced) study - protocol for a randomized controlled trial | Code 3 |
| Rao, V.N. | Adiposity and Incident Heart Failure and its Subtypes: MESA (Multi-Ethnic Study of Atherosclerosis) | Code 1 |
| Rayner, J.J. | Myocardial Energetics in Obesity: Enhanced ATP Delivery Through Creatine Kinase with Blunted Stress Response | Code 1 |
| Rayner, J.J. | Very low calorie diets are associated with transient ventricular impairment before reversal of diastolic dysfunction in obesity | Code 3 |
| Rebuffe-Serive, M. | Biobehavioral effects of weight cycling | Code 1 |
| Register, T.C. | Relationships between serum adiponectin and bone density, adiposity and calcified atherosclerotic plaque in the African American-Diabetes Heart Study | Code 2 |
| Renzo, L.D. | Effects of dark chocolate in a population of normal weight obese women: A pilot study | Code 3 |
| Resnick, L.M. | Pulse waveform analysis of arterial compliance: Relation to other techniques, age, and metabolic variables | Code 1 |
| Rhéaume, C. | Low cardiorespiratory fitness levels and elevated blood pressure: What is the contribution of visceral adiposity? | Code 2 |
| Ribeiro dos Santos, V. | Predictive capacity of anthropometric indicators for abdominal fat in the oldest old | Code 2 |
| Ribeiro-Filho, F.F. | Two-hour insulin determination improves the ability of abdominal fat measurement to identify risk for the metabolic syndrome | Code 2 |
| Richelsen, B. | Lipoprotein lipase activity in muscle tissue influenced by fatness, fat distribution and insulin in obese females | Code 1 |
| Rider, O.J. | Determinants of left ventricular mass in obesity; A cardiovascular magnetic resonance study | Code 1 |
| Rijzewijk, L.J. | Myocardial steatosis is an independent predictor of diastolic dysfunction in type 2 diabetes mellitus | Code 1 |
| Robbins, N. | Computed tomographic quantification of periaortic adipose tissue volume as a correlate of cardiovascular disease | Code 3 |
| Rodriguez-Granillo, G.A. | Pericardial and visceral, but not total body fat, are related to global coronary and extra-coronary atherosclerotic plaque burden | Code 1 |
| Rolland, Y. | Body-composition predictors of mortality in women aged >= 75 y: data from a large population-based cohort study with a 17-y follow-up | Code 3 |
| Romero-Acevedo, L. | Handgrip strength and lean mass are independently related to brain atrophy among alcoholics | Code 2 |
| Romero-Corral, A. | Modest visceral fat gain causes endothelial dysfunction in healthy humans | Code 1 |
| Rosenberg, K. | Automated ct biomarkers predict cardiovascular events and survival better than clinical parameters | Code 2 |
| Rosenkranz, S.K. | Effects of a high-fat meal on pulmonary function in healthy subjects | Code 1 |
| Rosito, G.A. | Pericardial fat, visceral abdominal fat, cardiovascular disease risk factors, and vascular calcification in a community-based sample: the Framingham Heart Study | Code 2 |
| Rothney, M.P. | Abdominal visceral fat measurement using dual-energy X-ray: association with cardiometabolic risk factors | Code 2 |
| Ruhl, C.E. | The association of low serum alanine aminotransferase activity with mortality in the us population | Code 3 |
| Ruppert, J. | Increased pericardial adipose tissue and cardiometabolic risk in patients with schizophrenia versus healthy controls | Code 2 |
| Ryan, A.S. | Metabolic Benefits of Prior Weight Loss with and without Exercise on Subsequent 6-Month Weight Regain | Code 1 |
| Ryan, A.S. | The insertion/deletion polymorphism of the ACE gene is related to insulin sensitivity in overweight women | Code 1 |
| Ryan, A.S. | Dietary restriction and walking reduce fat deposition in the midthigh in obese older women | Code 1 |
| Ryckman, E.M. | Visceral fat quantification in asymptomatic adults using abdominal CT: is it predictive of future cardiac events? | Code 2 |
| Ryu, J.E. | Relationship of intraabdominal fat as measured by magnetic resonance imaging to postprandial lipemia in middle-aged subjects | Code 1 |
| S. Farsijani | Body Composition by Computed Tomography vs Dual-Energy X-ray Absorptiometry: Long-Term Prediction of All-Cause Mortality in the Health ABC Cohort | Code 3 |
| S. H. Chou | Effects of Vitamin D3 Supplementation on Body Composition in the VITamin D and OmegA-3 TriaL (VITAL) | Code 1 |
| S. Horber, R | Hemostatic alterations linked to body fat distribution, fatty liver, and insulin resistance | Code 2 |
| S. Jongjirasiri | Relation of visceral adipose tissue to coronary artery calcium in Thai patients | Code 2 |
| S. Lim | Postprandial dyslipidemia after a standardized high-fat meal in BMI-matched healthy individuals, and in subjects with prediabetes or type 2 diabetes | Code 2 |
| S. Remmelzwaal | Sex-specific associations of body composition measures with cardiac function and structure after 8 years of follow-up | Code 1 |
| S. Saeed | The prevalence and covariates of stroke in khyber pakhtunkhwa; from a european perspective | Code 2 |
| S. Sarma | Elevated exercise blood pressure in middle-aged women is associated with altered left ventricular and vascular stiffness | Code 2 |
| Sadeghi, M. | Association of apolipoprotein B, apolipoprotein A, and the its ratio with body fat distribution | Code 2 |
| Sadeghi, M. | Abdominal fat distribution and serum lipids in patients with and without coronary heart disease | Code 5 |
| Salamat, M.R. | Anthropometric predictive equations for estimating body composition | Code 2 |
| Sam, A.H. | Circulating pancreatic polypeptide concentrations predict visceral and liver fat content | Code 1 |
| Sam, S. | Relationship of abdominal visceral and subcutaneous adipose tissue with lipoprotein particle number and size in type 2 diabetes | Code 1 |
| Samaras, K. | Effects of postmenopausal hormone replacement therapy on central abdominal fat, glycemic control, lipid metabolism, and vascular factors in type 2 diabetes: A prospective study | Code 1 |
| Samaras, K. | Independent genetic factors determine the amount and distribution of fat in women after the menopause | Code 1 |
| Samaropoulos, X.F. | A metabolically healthy obese phenotype in hispanic participants in the IRAS family study | Code 1 |
| Sánchez-Rodríguez, D. | Malnutrition according to ESPEN definition predicts long-term mortality in general older population: Findings from the EPIDOS study-Toulouse cohort | Code 3 |
| Sandeep, S. | Serum visfatin in relation to visceral fat, obesity, and type 2 diabetes mellitus in Asian Indians | Code 2 |
| Santanasto, A.J. | Body Composition Remodeling and Incident Mobility Limitations in African Ancestry Men | Code 1 |
| Santanasto, A.J. | Body Composition Remodeling and Mortality: The Health Aging and Body Composition Study | Code 3 |
| Santanasto, A.J. | Effects of changes in regional body composition on physical function in older adults: A pilot randomized controlled trial | Code 1 |
| Sarac, I. | Gender differences in VLDL1 and VLDL2 triglyceride kinetics and fatty acid kinetics in obese postmenopausal women and obese men | Code 1 |
| Sato, F. | Association of epicardial, visceral, and subcutaneous fat with cardiometabolic diseases | Code 2 |
| Saunders, T.J. | Sedentary Behaviour, Visceral Fat Accumulation and Cardiometabolic Risk in Adults: A 6-Year Longitudinal Study from the Quebec Family Study | Code 2 |
| Sawada, N. | Influence of visceral adiposity accumulation on adverse left and right ventricular mechanics in the community | Code 2 |
| Sawada, N. | The Significance of the Effect of Visceral Adiposity on Left Ventricular Diastolic Function in the General Population | Code 2 |
| Scherbakov, N. | Cachexia as a common characteristic in multiple chronic disease | Code 2 |
| Scherer, M. | Blood plasma lipidomic signature of epicardial fat in healthy obese women | Code 1 |
| Schlett, C.L. | Association between abdominal adiposity and subclinical measures of left-ventricular remodeling in diabetics, prediabetics and normal controls without history of cardiovascular disease as measured by magnetic resonance imaging: Results from the KORA-FF4 Study | Code 2 |
| Schoen, R.E. | Lack of association between adipose tissue distribution and IGF-1 and IGFBP-3 in men and women | Code 1 |
| Schols, A.M.W. | Body composition and mortality in chronic obstructive pulmonary disease | Code 3 |
| Schousboe, J.T. | Central Obesity and Visceral Adipose Tissue Are Not Associated With Incident Atherosclerotic Cardiovascular Disease Events in Older Men | Code 1 |
| Schouten, F. | Increases in central fat mass and decreases in peripheral fat mass are associated with accelerated arterial stiffening in healthy adults: the Amsterdam Growth and Health Longitudinal Study | Code 1 |
| Schutte, S. | A 12-wk whole-grain wheat intervention protects against hepatic fat: the Graandioos study, a randomized trial in overweight subjects | Code 4 |
| Seimon, R.V. | Less waste on waist measurements: Determination of optimal waist circumference measurement site to predict visceral adipose tissue in postmenopausal women with obesity | Code 1 |
| Shabestari, A.A. | Abdominal fat sonographic measurement compared to anthropometric indices for predicting the presence of coronary artery disease | Code 2 |
| Shah, R.V. | Association of multiorgan computed tomographic phenomap with adverse cardiovascular health outcomes: The Framingham Heart Study | Code 8 |
| Shah, R.V. | Abdominal fat radiodensity, quantity and cardiometabolic risk: The Multi-Ethnic Study of Atherosclerosis | Code 1 |
| Shay, C.M. | Regional adiposity and risk for coronary artery disease in type 1 diabetes: Does having greater amounts of gluteal-femoral adiposity lower the risk? | Code 2 |
| Shea, J.L. | Body fat percentage is associated with cardiometabolic dysregulation in BMI-defined normal weight subjects | Code 3 |
| Shea, J.L. | The Prevalence of Metabolically Healthy Obese Subjects Defined by BMI and Dual-Energy X-Ray Absorptiometry | Code 3 |
| Shea, M.K. | The effect of pioglitazone and resistance training on body composition in older men and women undergoing hypocaloric weight loss | Code 1 |
| Shiina, Y. | Relationships between the visceral fat area on CT and coronary risk factor markers | Code 1 |
| Shimabukuro, M. | Epicardial adipose tissue volume and adipocytokine imbalance are strongly linked to human coronary atherosclerosis | Code 5 |
| Shimamoto, Y. | Is visceral fat really a coronary risk factor?: A multi-detector computed tomography study | Code 1 |
| Shroff, R. | Young obese women with polycystic ovary syndrome have evidence of early coronary atherosclerosis | Code 1 |
| Sicari, R. | Pericardial rather than epicardial fat is a cardiometabolic risk marker: An MRI vs echo study | Code 1 |
| Sironi, A.M. | Impact of increased visceral and cardiac fat on cardiometabolic risk and disease | Code 1 |
| Sivam, S. | Effects of 8 weeks of continuous positive airway pressure on abdominal adiposity in obstructive sleep apnoea | Code 1 |
| Slinde, F. | Body composition and energy expenditure in patients with chronic obstructive pulmonary disease | Code 3 |
| Smith, S.R. | Orlistat 60 mg reduces visceral adipose tissue: a 24-week randomized, placebo-controlled, multicenter trial | Code 1 |
| Someya, Y. | Skeletal muscle function and need for long-term care of urban elderly people in Japan (the Bunkyo Health Study): A prospective cohort study | Code 1 |
| Sornay-Rendu, E. | Muscle mass is associated with incident fracture in postmenopausal women: The OFELY study | Code 1 |
| Spahillari, A. | The association of lean and fat mass with all-cause mortality in older adults: The Cardiovascular Health Study | Code 3 |
| Spauwen, P.J.J. | Associations of fat and muscle tissue with cognitive status in older adults: The AGES-Reykjavik Study | Code 2 |
| Stadnik, A.M.W. | The effect of body fat percentage and body fat distribution on skin surface temperature with infrared thermography | Code 1 |
| Staten, M.A. | Measurement of fat distribution by magnetic resonance imaging | Code 1 |
| Stewart, P.M. | Cortisol metabolism in human obesity: impaired cortisone-->cortisol conversion in subjects with central adiposity | Code 3 |
| Styer, A.K. | Building the bridge between lifestyle intervention, physical function, and cardiovascular risk in middle-aged women: Lessons learned from the Women on the Move Through Activity and Nutrition study | Code 2 |
| Svensson, J. | Two-month treatment of obese subjects with the oral growth hormone (GH) secretagogue MK-677 increases GH secretion, fat-free mass, and energy expenditure | Code 4 |
| Szulc, P. | Rapid loss of appendicular skeletal muscle mass is associated with higher all-cause mortality in older men: The prospective MINOS study | Code 3 |
| T. Colonetti | Whey protein and vitamin D supplementation in institutionalized older adults: a randomized trial | Code 4 |
| T. Nath | Body fat predicts exercise capacity in persons with Type 2 Diabetes Mellitus: A machine learning approach | Code 3 |
| Tabara, Y. | Association of hematological parameters with insulin resistance, insulin sensitivity, and asymptomatic cerebrovascular damage: The J-SHIP and toon health study | Code 2 |
| Tachibana, M. | Measurement of epicardial fat thickness by transthoracic echocardiography for predicting high-risk coronary artery plaques | Code 2 |
| Tahara, N. | Clinical and biochemical factors associated with area and metabolic activity in the visceral and subcutaneous adipose tissues by FDG-PET/CT | Code 2 |
| Taira, K. | Delayed post-prandial lipid metabolism in subjects with intra-abdominal visceral fat accumulation | Code 1 |
| Talbot, L.S. | Metabolic risk factors and Posttraumatic stress disorder: The role of sleep in young, healthy adults | Code 2 |
| Tamura R. | Association of pericardial fat volume measured on cardiac computed tomography with coronary risk factors and severity of coronary artery disease in patients with moderate coronary risk | Code 6 |
| Tanaka, K. | Target value of intraabdominal fat area for improving coronary heart disease risk factors | Code 4 |
| Tanaka, T. | Impact of abdominal fat distribution, visceral fat, and subcutaneous fat on coronary plaque scores assessed by 320-row computed tomography coronary angiography | Code 2 |
| Tang, A. | Insulin-sensitive overweight/obese individuals remain as insulin sensitive and normotensive as lean subjects over 6 years | Code 1 |
| Tchernof, A. | Impaired capacity to lose visceral adipose tissue during weight reduction in obese postmenopausal women with the Trp64Arg beta3-adrenoceptor gene variant | Code 1 |
| Tchernof, A. | Weight loss reduces C-reactive protein levels in obese postmenopausal women | Code 2 |
| Terry, J.G. | Association of smoking with abdominal adipose deposition and muscle composition in Coronary Artery Risk Development in Young Adults (CARDIA) participants at mid-life: A population-based cohort study | Code 1 |
| Tochikubo, O. | Improvement of multiple coronary risk factors in obese hypertensives by reduction of intra-abdominal visceral fat | Code 4 |
| Torres, T. | Epicardial adipose tissue and coronary artery calcification in psoriasis patients | Code 1 |
| Torres, T. | Complement C3 as a marker of cardiometabolic risk in psoriasis | Code 2 |
| Torriani, M. | Compartmental neck fat accumulation and its relation to cardiovascular risk and metabolic syndrome | Code 2 |
| Toss, F. | Abdominal and gynoid adiposity and the risk of stroke | Code 1 |
| Toss, F. | Body composition and mortality risk in later life | Code 3 |
| Totsikas, C. | Cardiorespiratory fitness determines the reduction in blood pressure and insulin resistance during lifestyle intervention | Code 1 |
| Tournadre, A. | Changes in body composition and metabolic profile during interleukin 6 inhibition in rheumatoid arthritis | Code 1 |
| Toyama, Y. | Impact of obstructive sleep apnea on liver fat accumulation according to sex and visceral obesity | Code 2 |
| Trémollieres, F.A. | Association of cardiovascular risk factors with intima-media thickness of the carotid arteries in early postmenopausal women | Code 1 |
| Treuth, M.S. | Estimating intraabdominal adipose tissue in women by dual-energy X-ray absorptiometry | Code 1 |
| Trikudanathan, S. | Association of female reproductive factors with body composition: the Framingham Heart Study | Code 2 |
| Tsaban, G. | Dynamics of intrapericardial and extrapericardial fat tissues during long-term, dietary-induced, moderate weight loss | Code 1 |
| Tsushima, H. | Association of epicardial and abdominal visceral adipose tissue with coronary atherosclerosis in patients with a coronary artery calcium score of zero | Code 2 |
| Ueda, Y. | Association between the presence or severity of coronary artery disease and pericardial fat, paracardial fat, epicardial fat, visceral fat, and subcutaneous fat as assessed by multi-detector row computed tomography | Code 5 |
| Ueno, S. | Association of neurological diseases with metabolic syndrome among out-patients | Code 5 |
| Utsunomiya, H. | Insulin resistance and subclinical abnormalities of global and regional left ventricular function in patients with aortic valve sclerosis | Code 2 |
| Vakhlamov, V.A. | Rationale for the use of new methods of investigation of metabolic syndrome in the diagnosis and treatment of patients with broncho-obstructive diseases | Code 1 |
| Van Aller, C. | Sarcopenic obesity and overall mortality: Results from the application of novel models of body composition phenotypes to the National Health and Nutrition Examination Survey 1999-2004 | Code 3 |
| Van Berendoncks, A.M. | Abnormal longitudinal peak systolic strain in asymptomatic patients with type I diabetes mellitus | Code 2 |
| Van Borst, B.D. | The influence of abdominal visceral fat on inflammatory pathways and mortality risk in obstructive lung disease | Code 5 |
| van den Borst, B. | Central fat and peripheral muscle: partners in crime in chronic obstructive pulmonary disease | Code 2 |
| Van Hout, M.J.P. | The effect of abdominal obesity on cardiovascular function and disease: a population based magnetic resonance imaging study of the uk biobank | Code 2 |
| VanWagner, L.B. | Nonalcoholic fatty liver disease and measures of early brain health in middle-aged adults: The CARDIA study | Code 2 |
| Vargas-Alarcón, G. | Interleukin-17A gene haplotypes are associated with risk of premature coronary artery disease in Mexican patients from the genetics of atherosclerotic disease (GEA) study | Code 5 |
| Vatanparast, H. | DXA-derived Abdominal Fat Mass, Waist Circumference, and Blood Lipids in Postmenopausal Women | Code 2 |
| Vella, C.A. | Associations of abdominal intermuscular adipose tissue and inflammation: The Multi-Ethnic Study of Atherosclerosis | Code 2 |
| Vella, C.A. | Associations of Abdominal Muscle Area and Radiodensity with Adiponectin and Leptin: The Multiethnic Study of Atherosclerosis | Code 2 |
| Vella, C.A. | Associations of insulin resistance with cardiovascular risk factors and inflammatory cytokines in normal-weight Hispanic women | Code 2 |
| Vella, CA. | Skeletal muscle area and density are associated with lipid and lipoprotein cholesterol levels: The Multi-Ethnic Study of Atherosclerosis | Code 2 |
| Villeneuve, N. | Interrelationships between changes in anthropometric variables and computed tomography indices of abdominal fat distribution in response to a 1-year physical activity-healthy eating lifestyle modification program in abdominally obese men | Code 1 |
| Violanti, J.M. | The Buffalo Cardio-Metabolic Occupational Police Stress (BCOPS) pilot study: methods and participant characteristics | Code 1 |
| Volek, J.S. | Effects of an 8-week weight-loss program on cardiovascular disease risk factors and regional body composition | Code 1 |
| Von Eyben, F.E. | Smoking, low density lipoprotein cholesterol, fibrinogen and myocardial infarction before 41 years of age: A Danish case-control study | Code 1 |
| W. F. Pereira-Manfro | Association between visceral/subcutaneous adipose tissue ratio and plasma inflammatory markers and score for cardiovascular risk prediction in a Brazilian cohort: Pro-Saude Study | Code 2 |
| W. P. P. Thu | Blood pressure and adiposity in midlife Singaporean women | Code 2 |
| Walker, K.Z. | Effects of regular walking on cardiovascular risk factors and body composition in normoglycemic women and women with type 2 diabetes | Code 1 |
| Walton, C. | Relationships between insulin metabolism, serum lipid profile, body fat distribution and blood pressure in healthy men | Code 4 |
| Walton, C. | Body fat distribution, rather than overall adiposity, influences serum lipids and lipoproteins in healthy men independently of age | Code 1 |
| Wander, P.L. | Change in visceral adiposity independently predicts a greater risk of developing type 2 diabetes over 10 years in Japanese Americans | Code 1 |
| Wanderley, F.A.C. | Six-minute walk distance (6MWD) is associated with body fat, systolic blood pressure, and rate-pressure product in community dwelling elderly subjects | Code 2 |
| Wang, T.D. | Association of epicardial adipose tissue with coronary atherosclerosis is region-specific and independent of conventional risk factors and intra-abdominal adiposity | Code 2 |
| Warren, M. | The relation between visceral fat measurement and torso level -- is one level better than another? The Atherosclerosis Risk in Communities Study, 1990-1992 | Code 1 |
| Wassel, C.L. | Associations of Abdominal Muscle Area with 4-Year Change in Coronary Artery Calcium Differ by Ethnicity Among Post-Menopausal Women | Code 1 |
| Weber-Hamann, B. | Metabolic changes in elderly patients with major depression: Evidence for increased accumulation of visceral fat at follow-up | Code 1 |
| Weber-Hamann, B. | Hypercortisolemic depression is associated with increased intra-abdominal fat | Code 1 |
| Weber, M.A. | Assessment of metabolism and microcirculation of healthy skeletal muscles by magnetic resonance and ultrasound techniques | Code 3 |
| Wei, C. | Cohort profile: The Lanxi Cohort study on obesity and obesity-related non-communicable diseases in China | Code 2 |
| Weinstein, G. | Non-alcoholic fatty liver disease, liver fibrosis score and cognitive function in middle-aged adults: The Framingham Study | Code 2 |
| Wijnhoven, H.A.H. | Region-specific fat mass and muscle mass and mortality in community-Dwelling older men and women | Code 3 |
| Wiklund, P. | Abdominal and gynoid adipose distribution and incident myocardial infarction in women and men | Code 3 |
| Wildman, R.P. | Subcutaneous adipose tissue in relation to subclinical atherosclerosis and cardiometabolic risk factors in midlife women | Code 1 |
| Williams, M.J. | Regional fat distribution in women and risk of cardiovascular disease | Code 2 |
| Wilman, H.R. | Genetic studies of abdominal MRI data identify genes regulating hepcidin as major determinants of liver iron concentration | Code 3 |
| Wilson, G.A. | HIIT Improves Left Ventricular Exercise Response in Adults with Type 2 Diabetes | Code 3 |
| Wilson, J.P. | Ratio of Trunk to Leg Volume as a New Body Shape Metric for Diabetes and Mortality | Code 3 |
| Winfield, R.D. | Adipose tissue location and contribution to postinjury hypercoagulability | Code 5 |
| Witasp, A. | Inflammatory biomarker pentraxin 3 (PTX3) in relation to obesity, body fat depots and weight loss | Code 2 |
| Woods, J.L. | Immunological and nutritional factors in elderly people in low-level care and their association with mortality | Code 3 |
| Woods, J.L. | Weight loss in elderly women in low-level care and its association with transfer to high-level care and mortality | Code 3 |
| Wu, F.Z. | Differential impacts of cardiac and abdominal ectopic fat deposits on cardiometabolic risk stratification | Code 2 |
| Wysham, K.D. | Association of High Anti–Cyclic Citrullinated Peptide Seropositivity and Lean Mass Index With Low Bone Mineral Density in Rheumatoid Arthritis | Code 2 |
| Xia, M.F. | A indicator of visceral adipose dysfunction to evaluate metabolic health in adult Chinese | Code 1 |
| Y. M. Cheung | Precision of the Hologic Horizon A dual energy X-ray absorptiometry in the assessment of body composition | Code 2 |
| Y. Qi | Relationship of Visceral Adipose Tissue With Dilated Perivascular Spaces | Code 2 |
| Y. Shibata | Association Between Major Adverse Cardiovascular Events and the Ratio of Subcutaneous Fat Area to Visceral Fat Area in Patients Who Have Undergone Multidetector Row Computed Tomography | Code 5 |
| Y. Xu | The mediating role of the visceral fat area in the correlation between the serum osteocalcin levels and a prolonged QTc interval | Code 2 |
| Yamada, A. | Association of Visceral Fat and Liver Fat With Hyperuricemia | Code 2 |
| Yamamoto, S. | Visceral fat accumulation, insulin resistance, and elevated depressive symptoms in middle-aged Japanese men | Code 2 |
| Yamasaki, Y. | Improved lipid profiles are associated with reduced incidence of coronary vascular events in asymptomatic patients with type 2 diabetes and impaired myocardial perfusion | Code 3 |
| Yamashiro, K. | Visceral fat accumulation is associated with cerebral small vessel disease | Code 2 |
| Yeoh, A.J. | The Association Between Subcutaneous Fat Density and the Propensity to Store Fat Viscerally | Code 1 |
| Yim, J.E. | Intermuscular adipose tissue rivals visceral adipose tissue in independent associations with cardiovascular risk | Code 1 |
| Yim, J.Y. | Sagittal abdominal diameter is a strong anthropometric measure of visceral adipose tissue in the asian general population | Code 1 |
| Yoon, J.W. | Hyperglycemia is associated with impaired muscle quality in older men with diabetes: The Korean Longitudinal Study on Health and Aging | Code 2 |
| Yu, E.W. | Fecal microbiota transplantation for the improvement of metabolism in obesity: The FMT-TRIM double-blind placebo-controlled pilot trial | Code 4 |
| Yuichiro, Y. | Regional Fat Distribution and Blood Pressure Level and Variability: The Dallas Heart Study | Code 2 |
| Yukio, K. | Effect of Lactobacillus gasseri SBT2055 in fermented milk on abdominal adiposity in adults in a randomised controlled trial | Code 1 |
| Zafrir, B. | Prognostic impact of abdominal fat distribution and cardiorespiratory fitness in asymptomatic type 2 diabetics | Code 3 |
| Zaslavsky, O. | Association of dynamics in lean and fat mass measures with mortality in frail older women | Code 5 |
| Zemski, A.J. | Differences in visceral adipose tissue and biochemical cardiometabolic risk markers in elite rugby union athletes of Caucasian and Polynesian descen | Code 1 |
| Zhou, T. | Effects of visceral fat area and other metabolic parameters on stone composition in patients undergoing percutaneous nephrolithotomy | Code 1 |
| Zhu, L. | Left ventricular myocardial deformation: a study on diastolic function in the Chinese male population and its relationship with fat distribution | Code 1 |
| Zong, G. | Total and regional adiposity measured by dual-energy X-ray absorptiometry and mortality in NHANES 1999-2006 | Code 3 |

* Codes:

Code 1: Outcome not available

Code 2: Study design

Code 3: Not abdominal VAT, or not assessed by DXA, CT or MRI

Code 4: Duration < 1 year or not available

Code 5: Population characteristics

Code 6: Japanese language

Code 7: Full text of the study unavailable

Code 8: Outcome not assessed in relation to measured abdominal VAT

**Appendix 3**

**Table. Summary of baseline characteristics of included studies. Values expressed as proportions (%), unless otherwise specified**

| **Author, Year**  **Country**  **Study Period** | **Comorbidities**  **(%)** | |
| --- | --- | --- |
| Ballin 2021 (1)  Sweden  2012-2018 | **Angina pectoris:** 8.2  **Myocardial infarction:** 4.4  **Stroke:** 3.3  **DM**: 8.7  **Fracture:** 16.0  **Kidney failure:** 0.7  **Cancer:** 16.8 | |
| Britton 2013 (2)  USA  2002-2005 | **HTN:** 26.7  **DM:** 5.4 | |
| Chung 2020 (3)  South Korea  2007-2018 | **DM:** Controls 4.0, Deceased 12.8  **HTN:** Controls 12.8, Deceased 22.3  **Fatty liver:** Controls 37.6, Deceased 35.8 | |
| De Santana 2019 (4)  Brazil  2005-2012 | **DM:**  Women Controls 20.0, Deceased 37.3  Men Controls 15.1, Deceased 30.8  **HTN:**  Women Controls 67.9, Deceased 79.1  Men Controls 52.7, Deceased 60.0  **Hyperlipidemia:**  Women Controls 15.1, Deceased 11.9  Men Controls 6.5, Deceased 15.4  **Previous cardiovascular event:**  Women Controls 10.5, Deceased 26.9  Men Controls 14.7, Deceased 30.8 | |
| Katzmarzyk 2012 (5)  USA  1995-2008 | NA | |
| Koster 2015 (6)  Iceland  2002-2013 | **Type 2 DM:** Women 9.5, Men 15.3  **CAD:** Women 13.6, Men 31.2 | |
| Kuk 2006 (7)  USA  1995-1999 | **HTN:** Controls 14.9, Deceased 30.0  **High cholesterol:** Controls 25.8, Deceased 26.8  **High triglycerides:** Controls 13.4, Deceased 16.5  **Type 2 DM:** Controls 2.1, Deceased 11.3 | |
| McNeely 2012 (8)  USA  1983-2007 | NA | |
| Mongraw-Chaffin 2017 (9)  USA  2002-2013 | **HTN:**  Tertile 1: 33.0, Tertile 2: 49.0, Tertile 3: 57.0, MVF: 67.0  **Type 2 DM:**  Tertile 1: 7.1, Tertile 2: 12.0, Tertile 3: 20.0, MVF: 29.0 | |
| Murphy 2014 (10)  USA  1997-2011 | Independent cohort:  Health ABC study | **Comorbid conditions:**  Women  0: Q1 12.6, Q2 16.1, Q3 17.2, Q4 15.4, Q5 16.4  1: Q1 25.6, Q2 25.8, Q3 21.9, Q4 27.9, Q5 31.6  2: Q1 26.7, Q2 28.3, Q3 31.9. Q4 23.9, Q5 25.1  ≥3: Q1 35.0, Q2 29.7, Q3 29.0, Q4 32.9, Q5 26.9  Men  0: Q1 17.2, Q2 16.7, Q3 16.2, Q4 17.1, Q5 20.5  1: Q1 31.7, Q2 30.1, Q3 28.8, Q4 32.3, Q5 27.6  2: Q1 28.0, Q2 27.5, Q3 25.8, Q4 23.8, Q5 28.4  ≥3: Q1 23.1, Q2 25.7, Q3 29.2, Q4 26.8, Q5 23.5 |
|  | Independent cohort:  AGES-Reykjavik study | **Comorbid conditions:**  Women  0: Q1 8.4, Q2 10.4, Q3 10.4, Q4 14.4, Q5 13.2  1: Q1 42.9, Q2 38.1, Q3 38.3, Q4 40.7, Q5 37.8  2: Q1 24.4, Q2 27.4, Q3 28.9, Q4 24.4, Q5 29.5  ≥3: Q1 24.3, Q2 24.1, Q3 22.4, Q4 20.5, Q5 19.5  Men  0: Q1 8.4, Q2 9.5, Q3 10.9, Q4 13.1, Q5 18.4  1: Q1 28.6, Q2 34.6, Q3 33.6, Q4 36.7, Q5 32.7  2: Q1 24.5, Q2 21.9, Q3 24.3, Q4 24.0, Q5 21.5  ≥3: Q1 38.5, Q2 33.9, Q3 31.3, Q4 26.2, Q5 27.4 |
| Shil Hong 2015 (11)  South Korea  2005-2011 | **DM:**  Women 28.5, Q1 10.2, Q2 32.0, Q3 34.6, Q4 42.2  Men 35.8, Q1 21.9, Q2 34.0, Q3 44.3, Q4 43.8  **HTN:**  Women 70.3, Q1 66.4, Q2 68.8, Q3 69.3, Q4 75.0  Men 64.4, Q1 51.4, Q2 66.0, Q3 67.9, Q4 71.4  **Hx. of MI, PCI, CABG:**  Women 4.8, Q1 4.7, Q2 5.5, Q3 7.9, Q4 3.1  Men 4.8, Q1 1, Q2 5.7, Q3 4.7, Q4 8.7  **Hx. of angina:**  Women 6.0, Q1 8.6, Q2 2.3, Q3 6.3, Q4 8.6  Men 4.3, Q1 0, Q2, 7.5, Q3 1.9, Q4 7.6  **Hx. of stroke:**  Women 10.7, Q1 10.2, Q2 8.6, Q3 6.3, Q4 14.8  Men 10.7, Q1 9.5, Q2 12.3, Q3 6.6, Q4 12.4 | |

HTN: Hypertension; DM: Diabetes; NA: Not available; CAD: Coronary artery disease; MVF: Missing Visceral Fat; Hx: history; MI: Myocardial infarction; PCI: Percutaneous coronary intervention; CABG: Coronary artery bypass surgery.

**References**

1. Ballin M, Nordström P, Niklasson J, Nordström A. Associations of visceral adipose tissue and skeletal muscle density with incident stroke, myocardial infarction, and all-cause mortality in community-dwelling 70-year-old individuals: A prospective cohort study. J Am Heart Assoc (2021) 10(9):e020065-e.

2. Britton KA, Massaro JM, Murabito JM, Kreger BE, Hoffmann UM, Fox CS. Body fat distribution, incident cardiovascular disease, cancer, and all-cause mortality. J Am Coll Cardiol (2013) 62(10):921-925.

3. Chung GE, Heo NJ, Kim D, et al. Association between advanced fibrosis in fatty liver disease and overall mortality based on body fat distribution. J Gastroenterol Hepatol (2020) 35(1):90-96.

4. De Santana FM, Domiciano DS, Goncalves MA, et al. Association of appendicular lean mass, and subcutaneous and visceral adipose tissue with mortality in older Brazilians: the Sao Paulo ageing & health study. J Bone Miner Res (2019) 34(7):1264-1274.

5. Katzmarzyk PT, Mire E, Bouchard C. Abdominal obesity and mortality: the pennington center longitudinal study. Nutr Diabetes (2012) 2(8):e42.

6. Koster A, Murphy RA, Eiriksdottir G, et al. Fat distribution and mortality: the AGES-Reykjavik study. Obesity (2015) 23(4):893-897.

7. Kuk JL, Katzmarzyk PT, Nichaman MZ, Church TS, Blair SN, Ross R. Visceral fat is an independent predictor of all-cause mortality in men. Obesity (2006) 14(2):336-341.

8. McNeely MJ, Shofer JB, Leonetti DL, Fujimoto WY, Boyko EJ. Associations among visceral fat, all-cause mortality, and obesity-related mortality in Japanese Americans. Diabetes Care (2012) 35(2):296-298.

9. Mongraw-Chaffin M, Allison MA, Burke GL, et al. CT-derived body fat distribution and incident cardiovascular disease: The multi-ethnic study of atherosclerosis. J Clin Endocrinol Metab (2017) 102(11):4173-4183.

10. Murphy RA, Register TC, Shively CA, et al. Adipose tissue density, a novel biomarker predicting mortality risk in older adults. J Gerontol A Biol Sci Med Sci (2014) 69(1):109-117.

11. Shil Hong E, Khang AR, Roh E, et al. Counterintuitive relationship between visceral fat and all-cause mortality in an elderly Asian population. Obesity (2015) 23(1):220-227.

**Appendix 4a.** **Funding and conflict of interest of included studies**

| **Author,**  **Year**  **Country** | **Funding** | **Conflict of Interest** |
| --- | --- | --- |
| **Ballin 2021 (1)**  **Sweden** | This study was supported by the Swedish Research Council (grant 2016- 02589 to Dr P. Nordström). | None |
| **Britton 2013 (2)**  **USA** | This study was supported by the National Heart, Lung, and Blood Institute’s Framingham Heart Study (contract N01-HC- 25195). Dr. Britton was supported by a Research Career Development Award (K12 HL083786) from the National Heart, Lung, and Blood Institute. | None |
| **Chung 2020 (3)**  **South Korea** | None | None |
| **De Santana 2019 (4)**  **Brazil** | This study was not sponsored by any pharmaceutical company. The SPAH project was supported by grants from the Fundacao de Amparo e Pesquisa do Estado de Sao Paulo (FAPESP #03/09313‐0, #04/12694‐8, and #09/15346‐4; FAPESP #11/00411‐5 to DSD); Conselho Nacional de Ciencia e Tecnologia (CNPQ #300559/2009‐7 and #301805/2013‐0 to RMRP), Federico Foundation (to RMRP), and Coordenacao de Aperfeicoamento de Pessoal de Nıvel Superior (CAPES to DSD, JBL, and CPF). | None |
| **Katzmarzyk 2012 (5)**  **USA** | PK is supported, in part, by the Louisiana Public Facilities Authority Endowed Chair in Nutrition and CB is funded, in part, by the John W Barton, Sr Endowed Chair in Genetics and Nutrition. This work was partially supported by a NORC Center grant no. 2P30-DK072476-06 entitled ‘Nutritional Programming: Environmental and Molecular Interactions’ sponsored by NIDDK. The PCLS is registered at ClinicalTrials.gov (identifier NCT00959270). | None |
| **Koster 2015 (6)**  **Iceland** | This study has been funded by NIH contract N01-AG-12100, the NIA Intramural Research Program, Hjartavernd (the Icelandic Heart Association), and the Althingi (the Icelandic Parliament). The study is approved by the Icelandic National Bioethics Committee, VSN: 00-063. | None |
| **Kuk 2006 (7)**  **USA** | This study was supported, in part, by research grants from the NIH to S.N.B. (AG06945) and M.Z.N. and Michael J. LaMonte (HL62508) and from the Canadian Institutes of Health Research to R.R. (MT13448) | NA |
| **McNeely 2012 (8)**  **USA** | This work was supported by the National Institutes of Health (grants DK-77745, DK-31170, HL-49293, and DK- 02654) and by facilities and services provided by the Diabetes and Endocrinology Research Center (DK-17047), Clinical Nutrition Re- search Unit (DK-35816), and General Clinical Research Center (RR-00037) at the University of Washington. VA Puget Sound provided support for Dr. Boyko’s involvement in this research. | None |
| **Mongraw-Chaffin 2017 (9)**  **USA** | This study was supported by contracts HHSN268201500003I, N01-HC-95159, N01-HC-95160, N01- HC-95161, N01-HC-95162, N01-HC-95163, N01-HC-95164, N01-HC-95165, N01-HC-95166, N01-HC-95167, N01-HC- 95168, N01-HC-95169, and HL088451 from the National Heart, Lung, and Blood Institute (NHLBI) and UL1-TR- 000040 and UL1-TR-001079 from the National Center for Research Resources.M.M.-C. was supported by training grant NHLBI 5T32HL007261-34. | None |
| **Murphy 2014 (10)**  **USA** | This study was supported by National Institutes of Health (N01-AG-6-2101, N01-AG-6-2103, N01-AG-6-2106; R01-AG28050, R01-AG28641, R01-HL39789, R01-NR-12459) and the Pepper Older Americans for Independence Center (P30 AG21332). This research was supported in part by the Intramural Research Program of the National Institutes of Health, National Institute on Aging. R.A.M. is supported by a Banting Postdoctoral Fellowship | NA |
| **Shil Hong 2015 (11)**  **South Korea** | This study was supported by Seoul National University Bundang Hospital (B-1304/198-110 and B-1304/198-109) and a grant of the Korean Health Technology R&D Project, Ministry for Health, Welfare, & Family Affairs, Republic of Korea (Grant No. A092077). The funders had no role in study design, data collection and analysis, decision to publish, or preparation of the manuscript. | None |

NA: Not available.

**Appendix 4b. Detailed risk of bias assessment of cohort studies using the Newcastle-Ottawa Quality Tool (12)**

| **Author** | **Representative-ness of the exposed cohort** | **Selection of non-exposed cohort** | **Ascertainment of exposure** | **Demonstrate outcome not present at start** | **Comparability of cohorts on the basis of design or analysis controlled for confounders** | **Assessment of outcome** | **Was follow up long enough for outcomes to occur (years)** | **Adequacy of follow-up of cohorts** | **Overall Quality** |
| --- | --- | --- | --- | --- | --- | --- | --- | --- | --- |
| **Ballin 2021 (1)**  **Sweden** | This study was based on the HAI, which is a population- based prevention study conducted at a single research clinic in Umeå, Sweden.  🡪 1 star | Same cohort for exposed and non-exposed  🡪 1 star | VAT measured by DXA in the entire population  🡪 1 star | All alive at beginning of study.  🡪 1 star | Model adjusted for sex, smoking, alcohol consumption, education, income, marital status, total fat mass, LDL, fasting blood glucose, systolic blood pressure, previous stroke /Myocardial infarction/ angina pectoris, prescribed antihypertensives/ anticoagulants/ lipid- lowering agents, moderate- to- vigorous physical activity and muscle density  🡪 2 stars | Mortality data were collected from the Swedish Cause of Death Register. These registers are maintained by the Swedish National Board of Health and Welfare.  🡪 1 star | Mean length of follow up 3.6y (range, 0.1– 6.6). In a sensitivity analysis, they excluded participants with follow-up < 1 y  🡪 1 star | Zero loss to follow-up  🡪 1 star | **Good**  **4-2-3** |
| **Britton 2013 (2)**  **USA** | Fremingham study represents Massachusett. But predominantly white individuals; population based  🡪 1 star | Same cohort for exposed and non-exposed  🡪 1 star | VAT measured by CT in the entire population  🡪 1 star | All alive at beginning of study. Excluded subjects with with CVD at baseline.  🡪 1 star | Model adjusted for age, sex, systolic blood pressure, diabetes, total cholesterol, HDL, current smoking, hypertension treatment, and BMI.  🡪 2 stars | Adjudication of outcome by three investigators  🡪 1 star | Median length of follow up 5y  🡪 1 star | No statement  🡪 0 stars | **Good**  4-2-2 |
| **Chung 2020 (3)**  **South Korea** | Participants from the Seoul National  University Hospital Healthcare System Gangnam Center; single center  🡪 1 star | Same cohort for exposed and non-exposed  🡪 1 star | VAT measured by CT in the entire population  🡪 1 star | All alive at beginning of study  🡪 1 star | Model adjusted for age, sex, diabetes, hypertension, fatty liver, subcutaneous adipose tissue area, and significant alcohol consumption.  🡪 2 stars | From the Korea National Statistical Office. Death certificates were identified with the use of the identification numbers assigned to subjects at birth.  🡪 1 star | Mean length of follow up 6.9 (2.7) y  🡪 1 star | No statement  🡪 0 stars | **Good**  4-2-2 |
| **De Santana 2019 (4)**  **Brazil** | São Paulo Ageing & Health (SPAH) Study, population based  🡪 1 star | Same cohort for exposed and non-exposed  🡪 1 star | VAT measured by DXA in the entire population  🡪 1 star | 10% had a CV event; given that we included up to 25% CV event at baseline  🡪 1 star | Model adjusted for age and other covariates and analysis was sex specific  🡪 2 stars | Death certificates available from the publicly available databases  🡪 1 star | Mean length of follow up 4.1 (1.1) y  🡪 1 star | 18% dropout  🡪 1 star | **Good**  4-2-3 |
| **Katzmarzyk 2012 (5)**  **USA** | Volunteers who have participated in a variety of clinical studies conducted at the Pennington Biomedical Research Center in Baton Rouge, Louisian; single center  🡪 1 star | Same cohort for exposed and non-exposed  🡪 1 star | VAT measured by CT in the entire population  🡪 1 star | All alive at beginning of study  🡪 1 star | Model adjusted for age, sex, exam year, smoking, alcohol, physical exercise and subcutaneous adipose tissue.  🡪 2 stars | Vital status of all participants and causes of death were determined by linkage to the National Death Index.  🡪 1 star | Mean length of follow up 9.1 (3.3) y  🡪 1 star | No statement  🡪 0 stars | **Good**  4-2-2 |
| **Koster 2015 (6)**  **Iceland** | Random sample of Reykjavik, population based  🡪 1 star | Same cohort for exposed and non-exposed  🡪 1 star | VAT measured by CT in the entire population  🡪 1 star | All alive at beginning of study  🡪 1 star | Model adjusted for age, sex and several other variables.  🡪 2 stars | Death from Icelandic National Roster  🡪 1 star | 11 years of follow-up with an average follow-up time of 8 years  🡪 1 star | No statement  🡪 0 stars | **Good**  4-2-2 |
| **Kuk 2006 (7)**  **USA** | Participants from the Cooper Clinic in Dallas, Texas; single clinic  🡪 0 stars | Same center but differring charachteristics  🡪 0 stars | VAT measured by CT in the entire population  🡪 1 star | All alive at beginning of study  🡪 1 star | Model adjusted for age, follow-up time, abdominal subcutaneous fat, and liver fat.  🡪 2 stars | National Death Index was used to identify potential deaths and cause, and official death certificates were obtained and were cross-referenced with the participant’s clinical record to confirm a match.  🡪 1 star | Mean length of follow up 2.2 (1.3) y  They state that it was short in their limitations  🡪 0 stars | No statement  🡪 0 stars | **Fair**  2-2-1 |
| **McNeely 2012 (8)**  **USA** | Recruitment for the Japanese Community Diabete Study; population-based  🡪 1 star | Same cohort for exposed and non-exposed  🡪 1 star | VAT measured by CT in the entire population  🡪 1 star | All alive at beginning of study  🡪 1 star | Model adjusted for age, sex and smoking  🡪 2 stars | Death identified through the National Death Index  🡪 1 star | Mean length of follow up  16.9 y  🡪 1 star | 71% had at least 3 visits; visits were 2.5 years apart.  🡪 1 star | **Good**  4-2-3 |
| **Mongraw-Chaffin 2017 (9)**  **USA** | MESA enrolled participants ages at six sites across the United States.  🡪 1 star | Same cohort for exposed and non-exposed  🡪 1 star | VAT measured by CT in 99% of participants. Participants whose VAT was not measured were assessed separately.  🡪 1 star | All alive at beginning of study.  Those with prevalent CVD were excluded from original MESA study  🡪 1 star | Model adjusted for age, sex, race /ethnicity, education, income, and smoking and subcutaneous fat.  🡪 2 stars | Information was collected from death certificates, medical records from hospitalizations, autopsy reports, interviews with participants, and, in the case of out-of-hospital deaths, interviews with or questionnaires administered to physicians, relatives, or friends  🡪 1 star | Mean length of follow-up 9.3 y  🡪 1 star | No statement  🡪 0 stars | **Good**  4-2-2 |
| **Murphy 2014 (10)**  **USA** | 1. Health ABC study recruited from Medicare beneficiaries in Memphis, Tennessee, and Pittsburgh, Pennsylvania. 2. AGES-Reykjavik is a single center study of participants living in Reykjavik Iceland; multi center   🡪 1 star | Same cohort for exposed and non-exposed  🡪 1 star | VAT measured by CT in the entire population  🡪 1 star | All alive at beginning of study  🡪 1 star | Model adjusted for age, race, study site, and education, and analysis was sex specific.  🡪 2 stars | Health ABC: Mortality was determined from death certificates, hospital records, and interview with next of kin. Causes of death were adjudicated by a central committee.  AGES-Reykjavik- Mortality was ascertained from the Icelandic National Roster, an adjudicatedregistry of deaths. Cause of death was ascertained from National Health System Records.  🡪 1 star | Health ABC total 14y of follow-up  AGES-Reykjavik range 4–9 y of follow-up  🡪 1 star | No statement  🡪 0 stars | **Good**  4-2-2 |
| **Shil Hong 2015 (11)**  **South Korea** | Stratified random sampling of subjects in Seongnam, South Korea, one of the satellite cities  of the Seoul Metropolitan district, population based  🡪 1 star | Same cohort for exposed and non-exposed  🡪 1 star | VAT measured by CT in the entire population  🡪 1 star | All alive at beginning of study  🡪 1 star | Model adjusted for age, sex, alcohol consumption, smoking status, and exercise habits, total fat mass  🡪 2 stars | Survival status of each individual and the cause of death were ascertained from the National Statistical Office. CVD-related deaths were identified as ICD-10 code I10-I11, I20-I25, or I6-1-64 for the cause of death.  🡪 1 star | Median length of follow-up 5.2 y (range 0.1-6.3)  🡪 1 star | Near-complete follow-up as data collected from National Statistical Office of South Korea. There would be minimal loss to follow-up (immigration or non-reported mortality); any loss to follow-up is likely to occur randomly. But actual number not listed.  🡪 1 star | **Good**  4-2-3 |

BMI: Body mass index; CVD: Cardiovascular disease; HDL: High-density lipoproteins; Health ABC: The Health, Aging, and Body Composition; LDL: Low-density lipoproteins; MESA: Multi-Ethnic Study of Atherosclerosis; VAT: Visceral adipose tissue

**References**

1. Ballin M, Nordström P, Niklasson J, Nordström A. Associations of visceral adipose tissue and skeletal muscle density with incident stroke, myocardial infarction, and all-cause mortality in community-dwelling 70-year-old individuals: A prospective cohort study. J Am Heart Assoc (2021) 10(9):e020065-e.

2. Britton KA, Massaro JM, Murabito JM, Kreger BE, Hoffmann UM, Fox CS. Body fat distribution, incident cardiovascular disease, cancer, and all-cause mortality. J Am Coll Cardiol (2013) 62(10):921-925.

3. Chung GE, Heo NJ, Kim D, et al. Association between advanced fibrosis in fatty liver disease and overall mortality based on body fat distribution. J Gastroenterol Hepatol (2020) 35(1):90-96.

4. De Santana FM, Domiciano DS, Goncalves MA, et al. Association of appendicular lean mass, and subcutaneous and visceral adipose tissue with mortality in older Brazilians: the Sao Paulo ageing & health study. J Bone Miner Res (2019) 34(7):1264-1274.

5. Katzmarzyk PT, Mire E, Bouchard C. Abdominal obesity and mortality: the pennington center longitudinal study. Nutr Diabetes (2012) 2(8):e42.

6. Koster A, Murphy RA, Eiriksdottir G, et al. Fat distribution and mortality: the AGES-Reykjavik study. Obesity (2015) 23(4):893-897.

7. Kuk JL, Katzmarzyk PT, Nichaman MZ, Church TS, Blair SN, Ross R. Visceral fat is an independent predictor of all-cause mortality in men. Obesity (2006) 14(2):336-341.

8. McNeely MJ, Shofer JB, Leonetti DL, Fujimoto WY, Boyko EJ. Associations among visceral fat, all-cause mortality, and obesity-related mortality in Japanese Americans. Diabetes Care (2012) 35(2):296-298.

9. Mongraw-Chaffin M, Allison MA, Burke GL, et al. CT-derived body fat distribution and incident cardiovascular disease: The multi-ethnic study of atherosclerosis. J Clin Endocrinol Metab (2017) 102(11):4173-4183.

10. Murphy RA, Register TC, Shively CA, et al. Adipose tissue density, a novel biomarker predicting mortality risk in older adults. J Gerontol A Biol Sci Med Sci (2014) 69(1):109-117.

11. Shil Hong E, Khang AR, Roh E, et al. Counterintuitive relationship between visceral fat and all-cause mortality in an elderly Asian population. Obesity (2015) 23(1):220-227.

12. Wells GA , Shea B , O'Connell D, et al. The newcastle-ottawa scale (NOS) for assessing the quality of nonrandomised studies in meta-analyses. <http://www.ohri.ca/programs/clinical_epidemiology/oxford.asp> [Accessed March 17, 2022].
